# A novel gene of *Kalanchoe daigremontiana* confers plant drought resistance

**DOI:** 10.1038/s41598-018-20687-5

**Published:** 2018-02-07

**Authors:** Li Wang, Chen Zhu, Lin Jin, Aihua Xiao, Jie Duan, Luyi Ma

**Affiliations:** 1 Key Laboratory for Silviculture and Conservation of the Ministry of Education, College of Forestry, Beijing Forestry University, Beijing, China; 2 Shanghai Center for Plant Stress Biology, Shanghai Institute for Biological Sciences, Chinese Academy of Sciences, Shanghai, China; 3 Institute of Chinese Medicinal Materials, Nanjing Agricultural University, Nanjing, China; 4 National Energy R&D Center for Non-food Biomass, Beijing, China

## Abstract

*Kalanchoe* (*K*.) *daigremontiana* is important for studying asexual reproduction under different environmental conditions. Here, we describe a novel *KdNOVEL41* (*KdN41*) gene that may confer drought resistance and could thereby affect *K. daigremontiana* development. The detected subcellular localization of a KdN41/Yellow Fluorescent Protein (YFP) fusion protein was in the nucleus and cell membrane. Drought, salt, and heat stress treatment in tobacco plants containing the *KdN41* gene promoter driving β-glucuronidase (*GUS*) gene transcription revealed that only drought stress triggered strong GUS staining in the vascular tissues. Overexpression (OE) of the *KdN41* gene conferred improved drought resistance in tobacco plants compared to wild-type and transformed with empty vector plants by inducing higher antioxidant enzyme activities, decreasing cell membrane damage, increasing abscisic acid (ABA) content, causing reinforced drought resistance related gene expression profiles. The 3,3′-diaminobenzidine (DAB) and nitroblue tetrazolium (NBT) staining results also showed less relative oxygen species (ROS) content in *KdN41*-overexpressing tobacco leaf during drought stress. Surprisingly, by re-watering after drought stress, *KdN41*-overexpressing tobacco showed earlier flowering. Overall, the *KdN41* gene plays roles in ROS scavenging and osmotic damage reduction to improve tobacco drought resistance, which may increase our understanding of the molecular network involved in developmental manipulation under drought stress in *K. daigremontiana*.

## Introduction

Plant growth relies on balancing propagation and survival in a rapidly changing habitat since the plant is a stationary system that cannot escape an inhospitable environment, unlike animals^1^. Thus, having a flexible growth system to adapt to a rapidly changing environment is the key in a variety of plant species^2–4^. Characterization of the regulatory mechanisms of plants under abiotic stresses (drought, salt, and temperature stress) increases our understanding by considering abiotic resistance in plants as a specific event during the developmental process^5–8^. Many transcription factor (TF) family genes and microRNA (miR) genes such as miR398 and miR393 have been shown to improve plant abiotic resistance by manipulating downstream stress resistance gene expression via hormone signaling^9,10^. In addition, many studies have also indicated that cross-talk between growth and stress hormone signaling can result in developmental arrest and enhancement of plant survival, allowing propagation of the species^11,12^.

*Kalanchoe* (*K*.) *daigremontiana* is a model plant for studying photosynthetic activity in crassulacean acid metabolism (CAM) species and plantlet morphogenesis along the dentate leaf margin^13,14^. As a CAM species, the *K. daigremontiana* succulent leaf structure shows impressive tolerance in drought and high-temperature environments^15^. It is of great interest to understand how plantlet morphogenesis is maintained while environmental stress continues to apply pressure. Previously, we observed an interesting scenario regarding an increased number of plantlets along the *K. daigremontiana* leaf margin under light drought stress compared to well-watered conditions, which allowed us to study the regulatory mechanisms between development and drought resistance. Different gene expression patterns in leaves between light drought stress and well-watered plants were identified by Suppression Subtractive Hybridization (SSH) technology^16^. Among genes with modulated expression, the *KdN41* gene, without a known function, was identified.

Here, we hypothesized that this gene may participate in drought stress signal perception, which could affect plant development in *K. daigremontiana*. In this study, the full cDNA and promoter sequences of *KdN41* were sequenced and the characteristics of putatively encoded proteins were analyzed using bioinformatics tools. The expression patterns of the *KdN41* gene were also examined in *K. daigremontiana* leaves under different hormone and drought stress treatments. The spatial expression pattern of the *KdN41* gene was monitored by β-glucuronidase (GUS) expression driven by the *KdN41* gene promoter during different abiotic stress conditions (drought, salt, and heat stress). Finally, tobacco plants (*Nicotiana tabacum*, NT) overexpressing *KdN41* were studied to evaluate the role of this gene in drought resistance.

## Results

### Antioxidant genes enrich in *K. daigremontiana* leaf under drought stress

Previously, SSH cDNA library were constructed among well watered *K. daigremontiana* leaves and leaves under light stress^16^. The library revealed different expressed genes induced by drought stress. Lacking a complete genome sequence in *K. daigremontiana*, 361 ESTs were annotated by employing four public databases, non-redundant protein database (Nr), non-redundant nucleotide database (Nt), Swiss-Prot protein database (Swiss-Prot), and Kyoto encyclopedia of genes and genomes database (KEGG). (Supplementary Fig. S1).

When compared with the COG database, all annotated ESTs were divided into 19 different functional classifications, excluding those poorly characterized or functional unknown ESTs (Supplementary Fig. S2a). Among these types, the first five categories were Translation, Ribosomal structure and biogenesis, Posttranslational modification, protein turnover, chaperones, Carbohydrate transport and metabolism, Energy production and conversion, Lipid transport and metabolism.

The go ontology (GO) databases were used to further classify the functions of the predicted genes. Approximately 315 ESTs were classified into three main categories: ‘biological process’, ‘cellular component’ and ‘molecular function’ (Supplementary Fig. S2b). Within the ‘cellular component’ category, a large number of ESTs were annotated as ‘integral component of membrane’, while the major groups within the ‘molecular function’ category were ‘ATP binding’ and ‘metal ion binding’. It’s worth to mention that in the ‘Biological process’ category, ‘oxidation-reduction process’ (27 ESTs) was the most abundant subcategories, among which antioxidant enzymes like peroxidase (POD), catalase (CAT), as well as NADP related genes were included (Supplementary Table S1).

Based on these data, *KdN41* was predicted to participate in the pathways of antioxidant activities to increase plant drought resistance.

### Clone and bioinformatics analysis of the *KdN41* gene

Based on the partial cDNA sequence of *KdN41*, the full cDNA sequence of *KdN41* was determined to be 884 bp after splicing the products of 3′ and 5′ RACE (rapid-amplification of cDNA ends) (Supplementary Fig. S3). The full sequence of the *KdN41* promoter was found to be 194 bp (from gDNA 5′ beginning to the start of ATG initiation codon), which was determined by the genome walking method.

According to NCBI ORF Finder analysis, the possible open-reading frame (ORF) sequence length for KdN41 protein is 450 bp, encoding 149 amino acids. Nucleotide BLAST and protein BLAST analyses revealed no highly similar match with other known genes in other plant species. The protein characteristics of KdN41 examined by the ProtParam tool showed that the putative protein contains 20 types of amino acids (chemical formula: C_713_H_1112_N_196_O_250_S_5_) with a 16.59-kD molecular mass and an isoelectric point of 4.60. According to the SWISS-Model prediction of KdN41 protein structure, the structure showed 3.50 and 11.31% similarity, respectively, to *Arabidopsis thaliana* Polyadenylate-binding protein 2 and *Schizosaccharomyces pombe* Polyadenylate-binding protein 2. Therefore, *KdN41* appears to be a novel gene based on both nucleotide and amino acid sequence BLAST analyses.

The key *cis*-acting elements of the *KdN41* gene promoter were predicted to be MBS (MYB TF binding site previously implicated in drought stress response), TC-rich repeats (stress response element), and P-box (a gibberellin-responding element) based on PlantCare analysis (Supplementary Fig. S4).

### *KdN41* gene expression patterns under different hormone and drought stress treatments

Based on the multiple hormone response elements presented in the *KdN41* gene promoter, we hypothesized that its expression may fluctuate under different hormone signaling and drought stress conditions. Therefore, *KdN41* expression profiles in *K. daigremontiana* leaves treated with 4 kinds of hormones [gibberellins (GA_3_), salicylic acid (SA), abscisic acid (ABA), or methyl jasmonate (MeJa)] and 4 different (16%, 12%, 8%, 4%) soil water contents respectively were analyzed using qRT-PCR.

**Figure 1:**
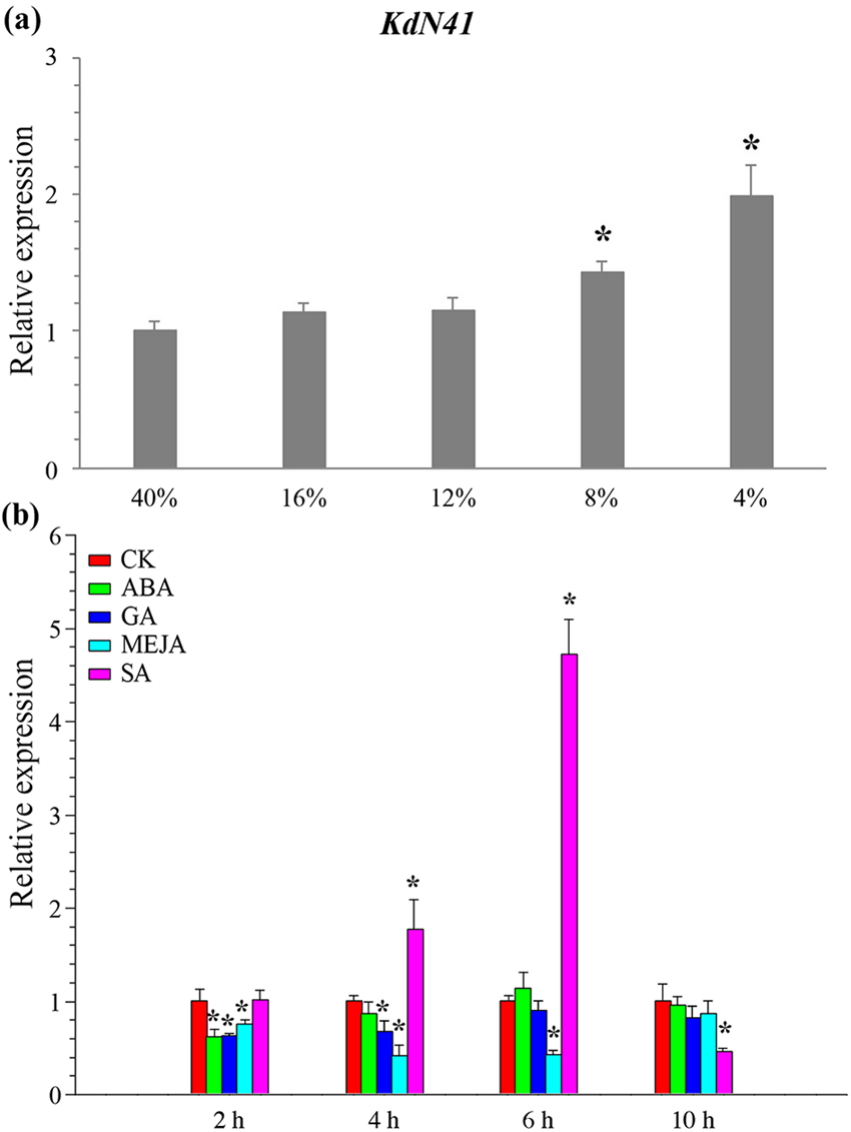
*KdN41* expression patterns in response to drought and different hormone treatments. (**a**) *KdN41*expression patterns in response to increasing drought stress. The horizontal axis represents relative soil water content, the water content of 40% is CK; (**b**) *KdN41*expression patterns in response to different hormone treatments. Abscisic acid (ABA), salicylic acid (SA) gibberellins (GA), methyl jasmonate (MeJa), and H_2_O (CK). *Indicated a significant difference when compared with CK (*p* < 0.05); Errors bars represented ± SD (standard deviations) of nine independent replications. The expression level of CK at 2 h was treated as reference and calculated as 1.

The *KdN41* gene expression showed no significant difference among leaves of *K. daigremontiana* plants under 16%, 12% and 40% (control group, CK) soil water contents (Fig. 1a). However, a slight increase in *KdN41* expression was observed in leaves of *K. daigremontiana* plants under 8% soil water content compared to CK (*P* < 0.05). Under 4% water content, the *KdN41* expression boosted almost 1 time more than CK (*P* < 0.05) (Fig. 1). Thus, the *KdN41* expression is sensitive to decreasing soil water content.

An extended effect of SA in upregulating the expression of the *KdN41* gene was more obvious than with other hormone treatments, suggesting that SA may be a key player in regulating this gene. After 6 h *KdN41* gene expression was upregulated significantly by ABA or GA treatment, but its expression was downregulated between 4 and 6 h in the MeJa treatment. *KdN41* upregulation was affected mainly by SA signaling and its downregulation was possibly affected by ABA, GA, or MeJa signaling (Fig. 1b).

### Sub-cellular localization of *KdN41* gene

We also used Yellow Fluorescent Protein (YFP) as a marker to monitor the sub-cellular localization of *KdN41* (using its ORF sequence) in tobacco leaf epidermal cells.

**Figure 2:**
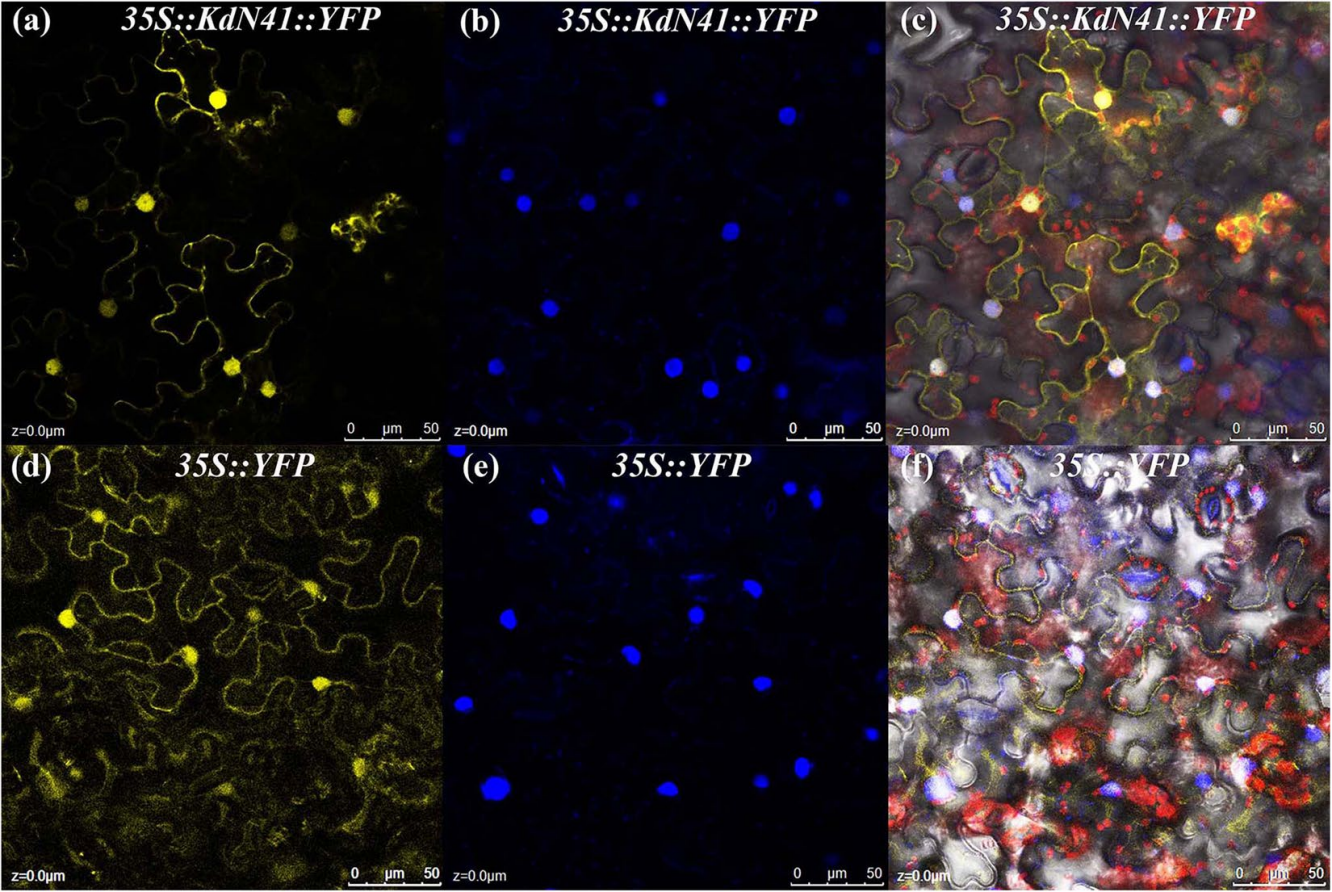
Sub-cellular localization of *KdN41* gene in tobacco epidermal cell. (**a**,**d**) 35*S::N41::YFP* and 35*S::YFP* signal detection; (**b**,**e**) DAPI staining signal detection; (**c**,**f**) merged image.

A strong signal of the KdN41-YFP fusion protein was primarily detected in the nucleus (Fig. 2a) and a weak signal was observed in the cell membrane (Fig. 2a), compared to the average signal in control samples (Fig. 2d).

Therefore, the *KdN41* gene expressed in the cell nucleus and membrane.

### *KdN41* gene expression pattern under drought stress signals

Since *KdN41* gene expression may be induced under stress conditions, according to its promoter sequence, we utilized a construct of the *KdN41* promoter driving *GUS* gene expression to determine the locations and conditions that induce *KdN41* gene expression. Next, three stress conditions (drought, salt, and heat) were used to test *KdN41* gene expression patterns.

**Figure 3:**
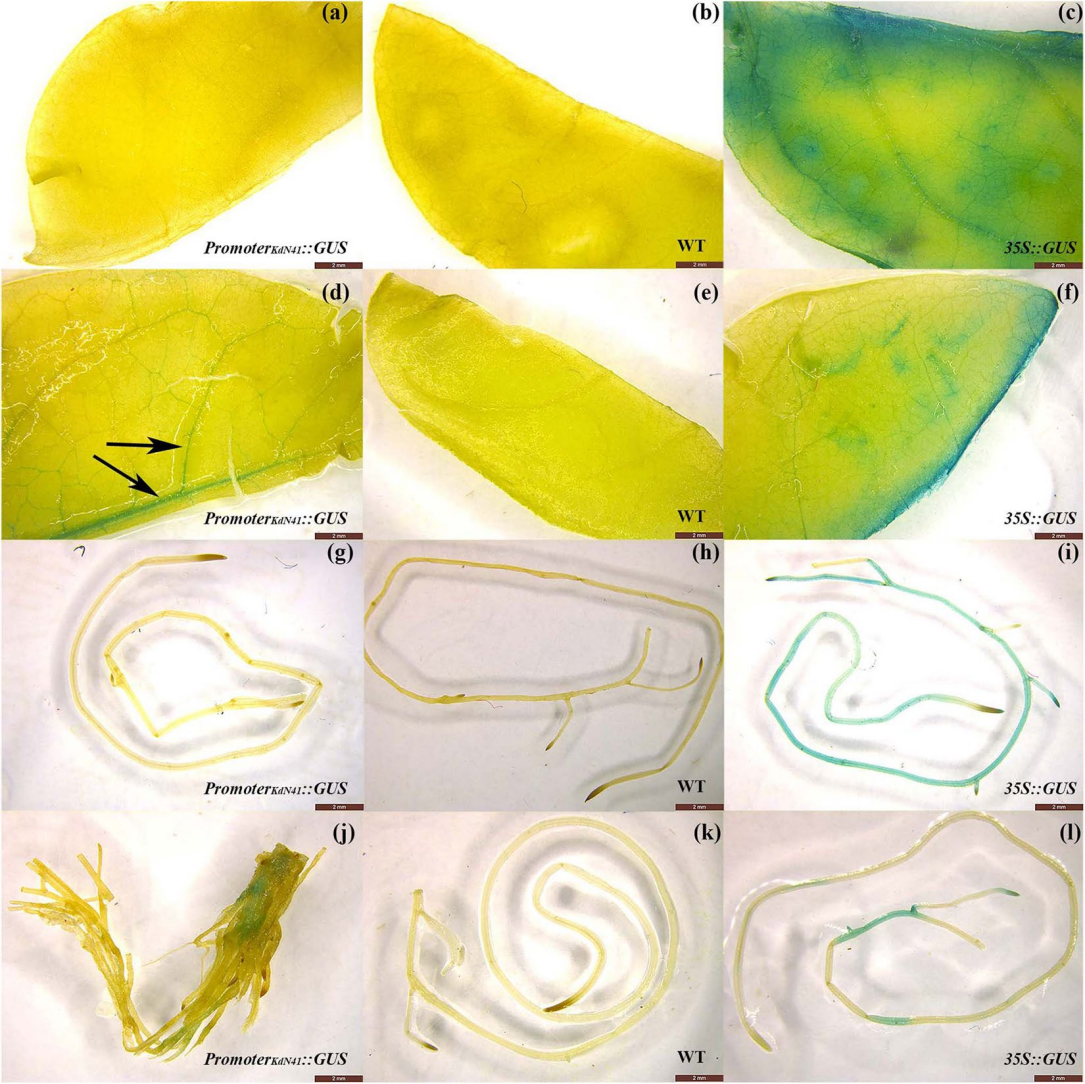
*KdN41* GUS staining analyses of *Promoter*_*KdN41*_*::GUS*, wild type (WT), and 35*S::GUS* (PC) tobacco plants after drought stress using 20% PEG. (**a**–**c**) leaf staining results with no drought treatment; (**d**–**f**) leaf staining results under drought treatment; (**g**–**i**) root staining results with no drought treatment; (**j**–**l**) root staining results under drought treatment; Black arrows mark GUS staining area in leaf vein of *Promoter*_*KdN41*_*::GUS* plants.

Interestingly, *KdN41* gene expression could not be detected in any tissue when no stress condition was applied, similar to wild-type (WT) plants (Fig. 3a and b). However, only 10 h after the onset of 20% (w/v) PEG6000 treatment (drought stress), *KdN41* gene expression detected by GUS staining was mainly observed in the leaf vein (Fig. 3d) compared to WT (Fig. 3e) and positive control (PC, transformed with 35*S::GUS*) plants (Fig. 3f). Weak GUS staining in the main root part (Fig. 3j) indicated faint *KdN41* gene expression compared to PC (Fig. 3l) and WT plants (Fig. 3k). After salt stress (Supplementary Fig. S5) or heat stress treatment (Supplementary Fig. S6), no GUS staining could be detected within the leaf vein or any root area compared to WT plants.

To summarize, *KdN41* gene expression was mostly silent during normal growth conditions, and only drought stress induced its high expression in the leaf vein.

### Over-expression of the *KdN41* gene confers drought stress resistance in tobacco

Given that the specific *KdN41* gene expression pattern under drought stress, this gene could play a role in tobacco drought stress tolerance. Therefore, the ability to resist drought stress (light drought, LD; medium drought, MD; severe drought, SD) was tested among *KdN41* over-expression (OE), WT, and negative control (NC, transformed with empty vector) plants.

**Figure 4:**
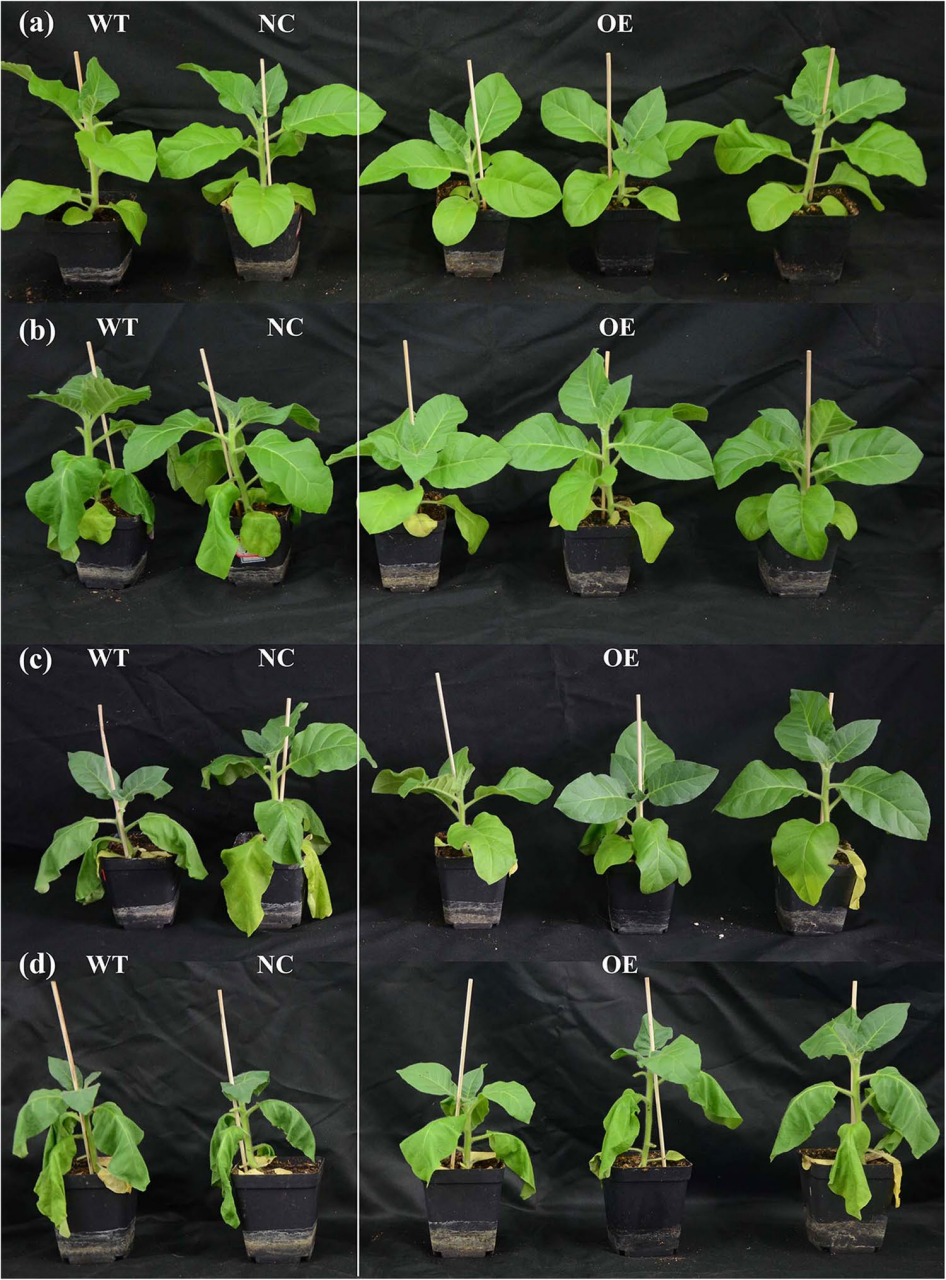
Different phenotypes in *35S::N41* (OE), wild type (WT) and NC (negative control) tobacco plants under increasing drought stress levels. (**a**) Plants with non-treatment; (**b**) plants under light drought (soil water content: 8–10%); (**c**) plants under medium drought (soil water content: 3–5%); (**d**) NC, OE and WT plants under severe drought (soil water content: <1%).

The phenotypes of OE, WT, and NC tobacco plants were compared under increasing drought treatment. OE plants (less leaf wilting) showed better drought resistance than WT, and NC plants, especially under SD stress (less than 1% soil water content) (Fig. 4).

Therefore, over-expression of *KdN41* gene improves drought stress resistance in tobacco.

### Over-expression of *KdN41* gene reduced reactive oxygen species (ROS) in tobacco leaves under drought stress

Drought is commonly considered as an induction of oxidative stress. The limited water availability often results in over-accumulation of ROS content such as hydrogen peroxide (H_2_O_2_) and super-oxygen ion (O_2_^−^), which is essential for plant to maintain homeostasis. Thus, we asked whether over-expression of *KdN41* gene could improve the elimination of excessive ROS content in tobacco leaves during drought stress. The 3,3′-diaminobenzidine (DAB) and nitroblue tetrazolium (NBT) staining methods were used to detect ROS localization in OE, WT, and NC tobacco leaves.

**Figure 5:**
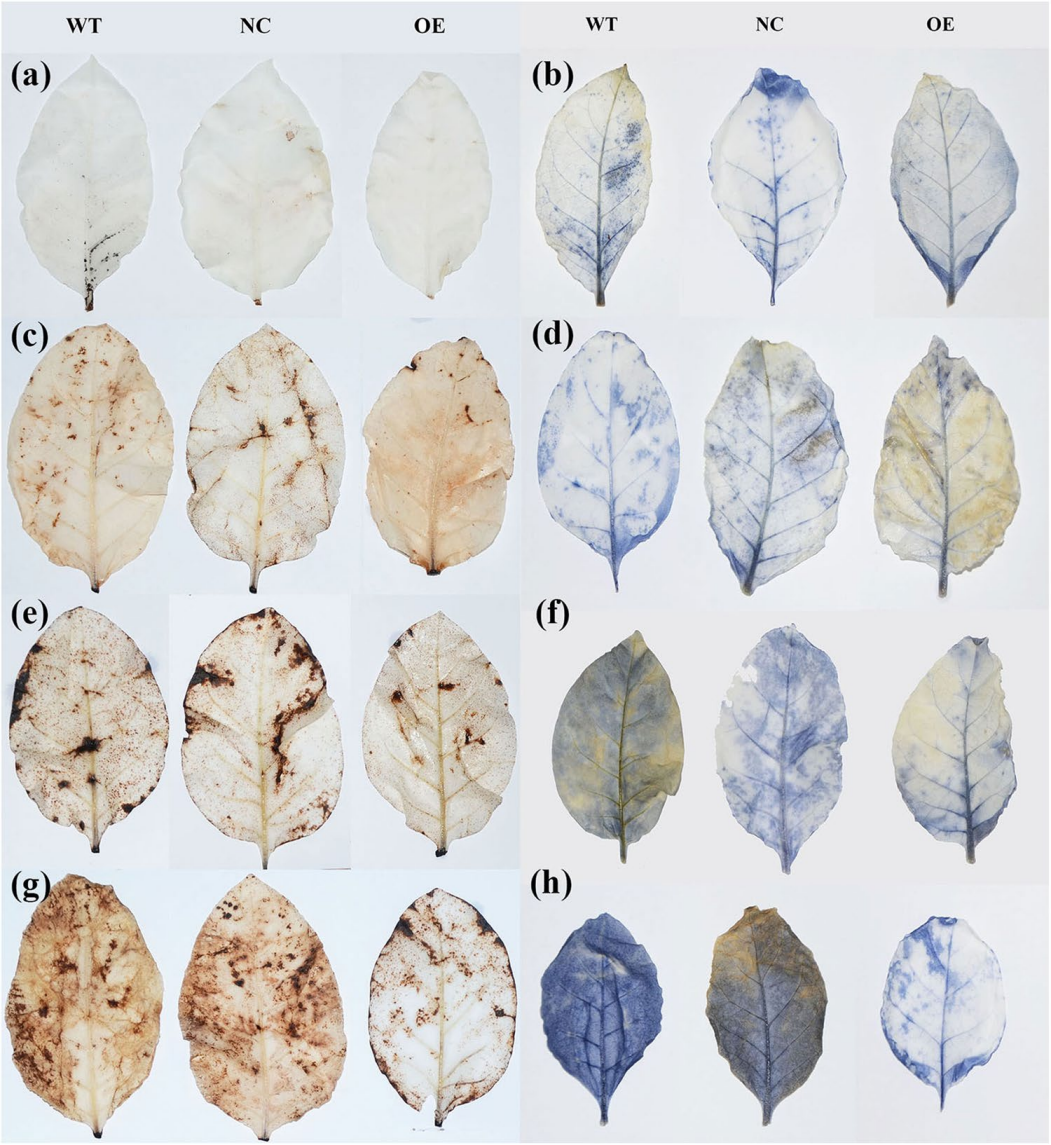
ROS staining of 35*S::N41* (OE), wild type (WT) and NC (negative control) tobacco plants under increasing drought stress levels. (**a**-**b**) Plants with non-treatment; (**b**-**c**) plants under light drought (soil water content: 8–10%); (**e**-**f**) plants under medium drought (soil water content: 3–5%); (**g**-**h**) NC, OE and WT plants under severe drought (soil water content: <1%).

Both DAB and NBT staining results showed small staining area of ROS in leaves of three genotypes under well watered condition (Fig. 5a,b). A slight increase in staining area of ROS was observed in WT and NC tobacco leaves (Fig. 5c,d) under LD stress condition (8–10% soil water content), however, no significant expansion in staining area of ROS was shown in OE tobacco leaves (Fig. 5c,d) under LD stress condition. Under MD stress (3–5% soil water content), a large staining area of ROS was showed in WT and NC tobacco leaves (Fig. 5e,f), while only limited increase could be observed in OE tobacco leaves (Fig. 5e,f). Under SD stress (less than 1% soil water content), almost the whole area of WT and NC tobacco leaves were stained (Fig. 5g,h), however, the staining area of ROS site in OE tobacco leaves was much smaller (Fig. 5g,h).

The well-maintained homeostasis of ROS content in OE tobacco leaves under drought stress suggested that the activities of antioxidant enzymes such as peroxidase (POD), catalase (CAT) were more robust in OE leaves than those of in WT and NC leaves. Thus, POD, CAT activities and H_2_O_2_, O_2_^−^ contents were also measured in OE, WT, and NC tobacco leaves.

**Figure 6:**
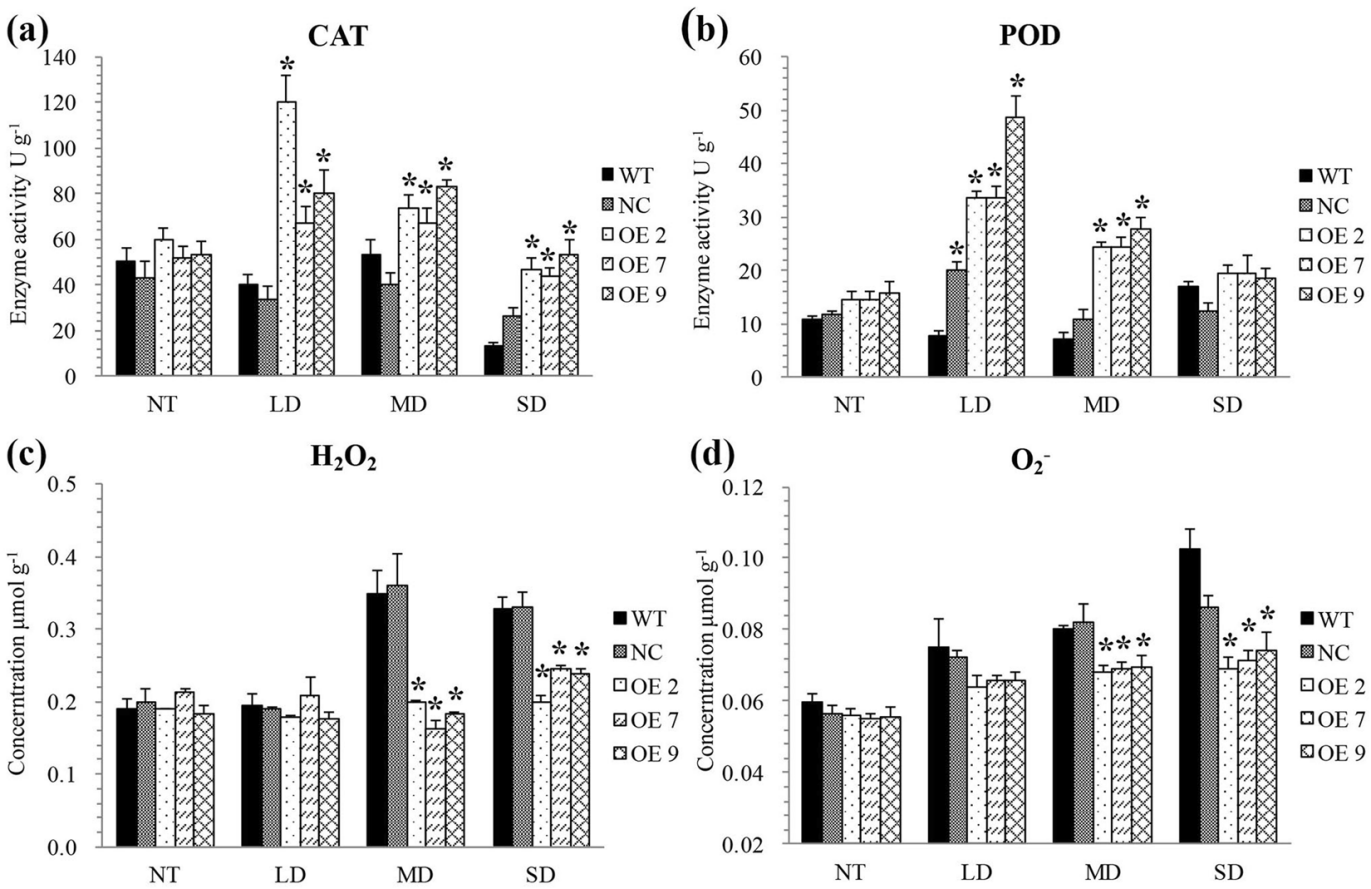
Changes of peroxidase (POD), catalase (CAT), H_2_O_2_ and O_2_^−^ content in 35*S::N41* (OE), wild type (WT) and NC (negative control) tobacco plants during increasing levels of drought stress, NT (non-treatment), LD (light drought), MD (medium drought), and SD (severe drought). Errors bars represented ± SD (standard deviations). *Indicated a significant difference compared to WT (Duncan’s multiple range test *p* < 0.05).

The POD and CAT activities showed no significant difference between three genotypes during well water condition (Fig. 6a,b), which was consistent with H_2_O_2_ and O_2_^−^ contents (Fig. 6c,d). A remarkable increase of both POD and CAT activities were found in OE tobacco leaves (*P* < 0.05) under LD and MD stress (Fig. 6a,b), leading to fewer contents of H_2_O_2_ and O_2_^−^ than those of in WT and NC under MD stress (*P* < 0.05) (Fig. 6c,d). Under SD stress, though POD and CAT activities went down in OE tobacco leaves, but was still higher than those of in WT and NC (*P* < 0.05), which was consistent with the lower contents of H_2_O_2_ and O_2_^−^ in OE tobacco leaves than those of in WT and NC leaves (*P* < 0.05) (Fig. 6c,d).

In summary, over-expression of *KdN41* gene increased antioxidant enzymes activities and eliminated excessive H_2_O_2_, O_2_^−^ contents in tobacco leaves.

### Over-expression of *KdN41* gene reduced osmotic damage in tobacco leaves under drought stress

The improved drought resistance of OE tobacco might also perform well in controlling osmotic damage and ABA signaling. Therefore, the electrolyte leakage (EL), Proline, MDA (Malondialdehyde) and ABA contents were tested in OE, WT and NC tobacco leaves.

**Figure 7:**
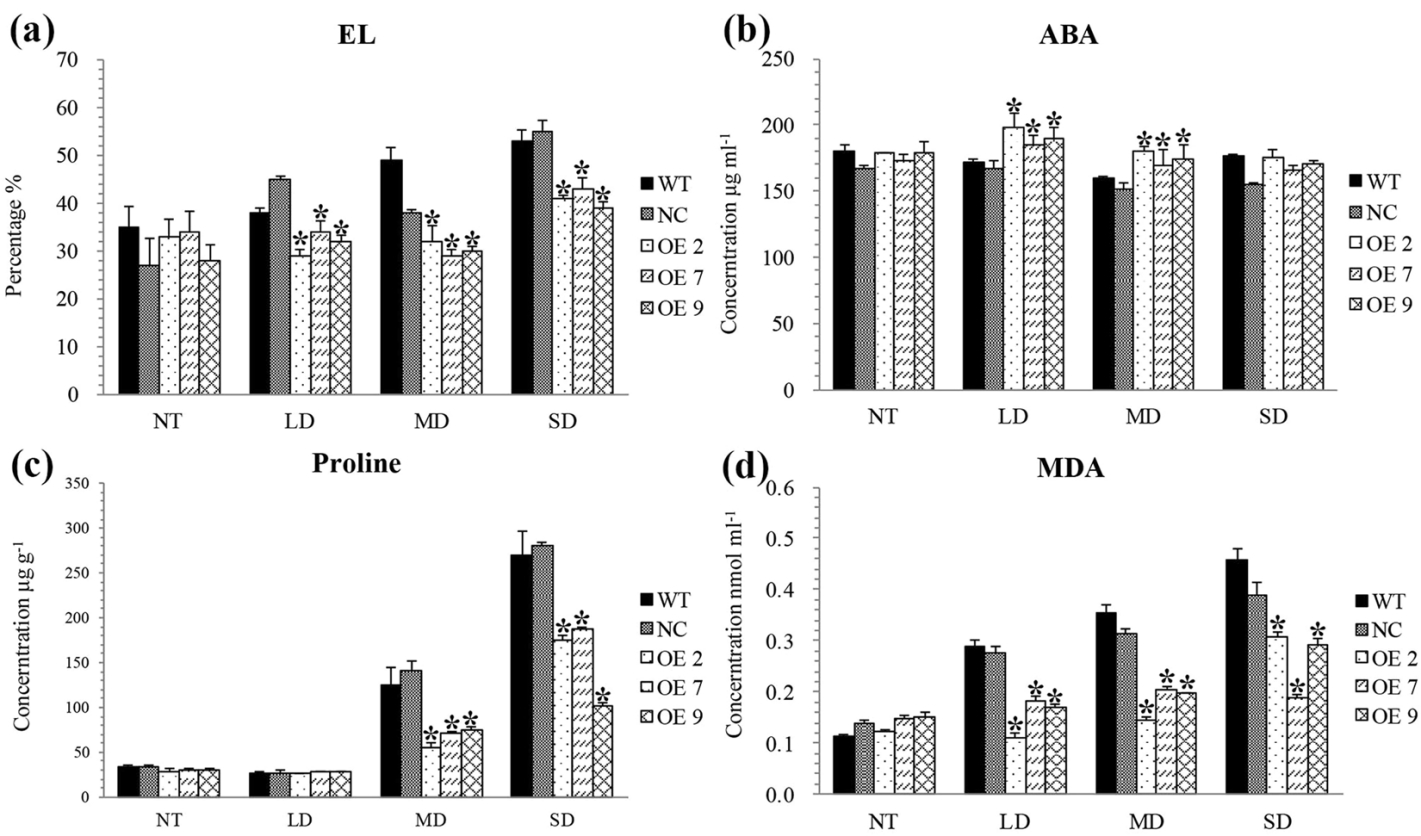
Electrolyte leakage (EL) activities, abscisic acid (ABA), Proline and malondialdehyde (MDA) content in 35*S::N41* (OE), wild type (WT) and NC (negative control) tobacco plants during increasing levels of drought stress, NT (non-treatment), LD (light drought), MD (medium drought), and SD (severe drought). Errors bars represented ± SD (standard deviations). *Indicated a significant difference when compared to WT (Duncan’s multiple range test *p* < 0.05).

In OE plants, EL, Proline and MDA results indicated better drought resistance and lower leaf cell membrane damage than WT and NC plants (Fig. 7a,c and d). The leaf ABA contents of OE plants slightly increased after drought treatment and was higher than those of WT and NC under LD and MD (Fig. 7b), which was consistent with the drought resistance phenotypes.

To summarize, over-expression of *KdN41* gene reduced osmotic damage in tobacco leaves under drought stress.

### Drought resistance related genes expression profiles in OE *KdN41* gene tobacco leaves under drought stress

Given that the improved drought resistance of *KdN41* gene OE plants, we also asked whether drought resistance related genes expression profiles were consistent with the phenotype. The relative expression of *KdN41*, *respiratory burst oxidase homolog D* (*RbohD*), *POD1*, *CAT*, *Superoxide Dismutase* (*SOD*) and *dehydration-responsive element binding like* (*DREB-like*) gene profiles in OE, WT, and NC tobacco leaves during drought stress were determined by RT-qPCR.

**Figure 8:**
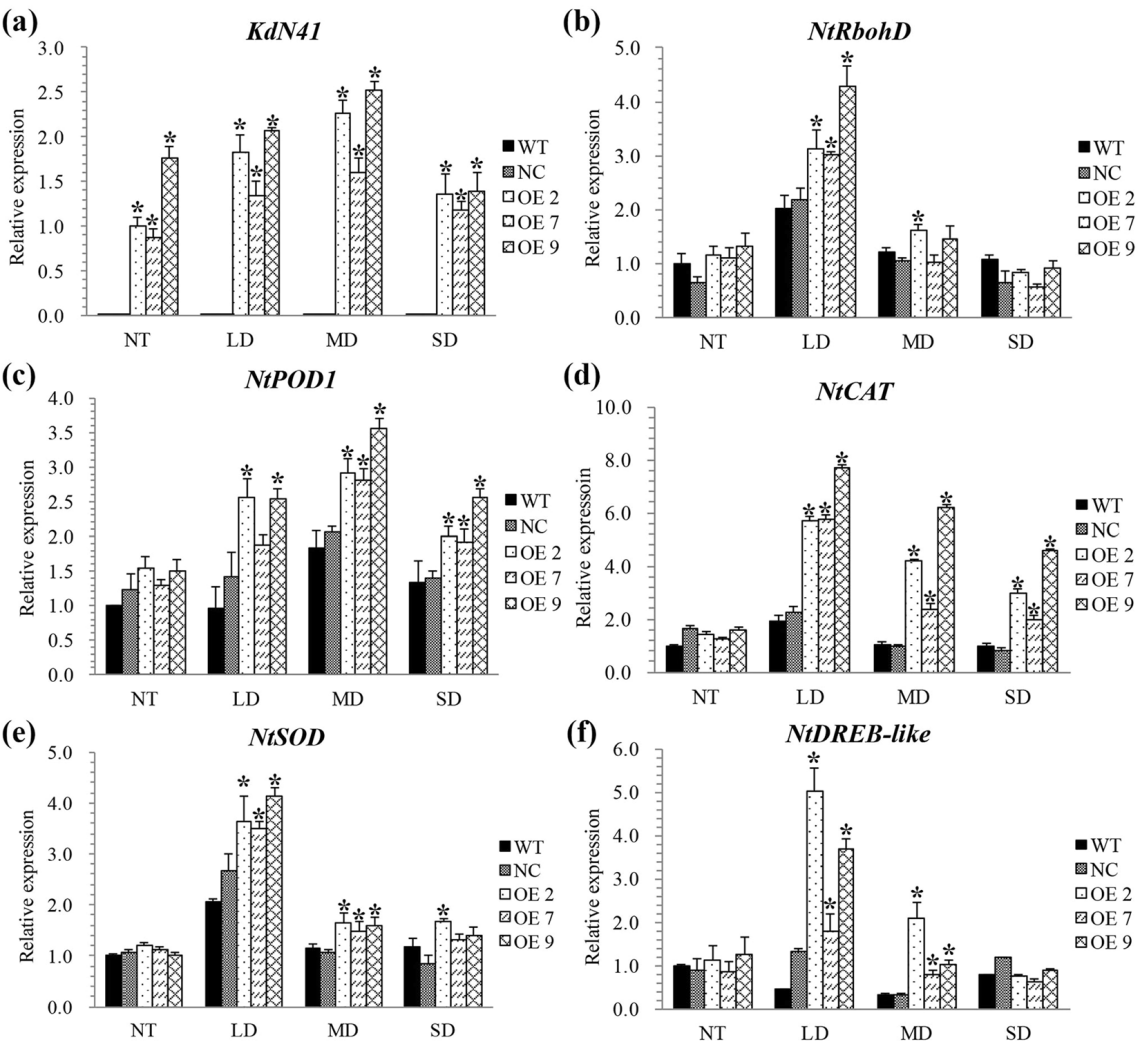
Relative expressions of drought resistance related genes in *35S::N41* (OE), wild type (WT) and NC (negative control) tobacco plants in response to increasing levels of drought stress, NT (non-treatment), LD (light drought), MD (medium drought), and SD (severe drought). Errors bars represented ± SD (standard deviations). *Indicated a significant difference compared to WT (Duncan’s multiple range test *p* < 0.05). The expression level of WT group under NT was treated as reference and calculated as 1.

The *KdN41* gene in OE leaves showed constitutive expression during well watered condition and drought stress (Fig. 8a). Mainly under LD stress, expressions of *NtRbohD*, *NtSOD* or *NtDREB-like* were found to be higher in OE than those of in WT and NC leaves (*P* < 0.05) (Fig. 8b,e and f). The *POD1* and *CAT* gene expressions were both strongly up-regulated in OE leaves during drought stress, and became significant higher than those of in WT and NC leaves under LD, MD, and SD (*P* < 0.05) (Fig. 8c,d).

In summary, over-expression of *KdN41* gene in tobacco resulted in reinforced antioxidant enzymes encoding genes expression and lower ROS synthesis gene (*RbohD*) expression during drought stress, which confers reduced oxidative damage in OE leaves.

### *KdN41* gene over-expression causes early flowering in tobacco

Surprisingly, the early-flowering phenomenon was observed in *KdN41*-expressing transgenic tobacco plants compared to WT and NC tobacco plants, after watering was restored post-drought treatment (Supplementary Fig. S7). In *KdN41*-expressing tobacco plants, the first flower bud formed at week 20. In the following week, half of the total plants under evaluation exhibited flowering. All plants under evaluation flowered by week 22. For WT and NC plants, no flower was observed until week 21. Moreover, it was not until week 24 that all plants of these two kinds of transgenic plants were observed with flower buds. Thus, the flowering time for OE plants was earlier by almost 20 days compared to WT and NC tobacco plants (Supplementary Table S2).

This early flowering phenotype of OE plants might indicate that this gene also plays a role in strengthening the developmental phase change post-drought stress.

## Discussion

The expression profiles of *KdN41* gene revealed by GUS staining under drought, salt, and heat stresses showed that this gene specifically responds to drought stress signaling, which is consistent with its appearance in our previous drought stress SSH cDNA library. In other studies, ROS have been repeatedly shown to be a key signal cascade mediating stress signaling^17^. We explored whether ROS could accompany the expression of *KdN41* gene, using DAB staining to visualize H_2_O_2_ localization in leaves under drought, salt, and heat stress (Supplementary Fig. S8 and S9). Under drought stress, the location of *KdN41* gene expression by GUS staining was observed (Fig. 3) to coincide with the localization of H_2_O_2_ by DAB staining (Supplementary Fig. S9). Under salt stress, the presence of H_2_O_2_ did not trigger *KdN41* gene expression. Therefore, *KdN41* gene expression may be independent of ROS signaling, and instead may be a specific drought stress signaling responder.

The short promoter of *KdN41* gene harboring multiple stress response elements suggests that this gene is tightly regulated by other gene networks. Thus, during normal growth conditions, the low expression of *KdN41* gene could play an important regulatory role in growth. Considering the *KdN41* expression patterns in response to drought signal, this gene may also function in tobacco salt and heat stress tolerance. To test this hypothesis, the ability to resist salt and heat was evaluated in OE, WT, and NC plants. During 7 days of continuous salt stress, a similar response was observed among OE, WT, and NC tobacco seedlings. The leaves of almost every plant turned from green to yellow, and no increased tolerance was observed after *KdN41* over-expression (Supplementary Fig. S10a). For the heat stress treatment, OE, WT, and NC tobacco seedlings were compared under a constant high temperature of 50 °C. After 7 h of heat stress, almost all OE, WT, and NC plants showed similar responses, with the leaves beginning to turn brown. No significant difference was observed between samples of OE and samples of the two other groups (Supplementary Fig. S10b). However, OE tobacco seedlings of similar developmental stage under PEG drought stress displayed better resistance phenotypes than WT and NC plants (Supplementary Fig. S11).

*KdN41* gene expression responded to drought stress signals, and over-expression of *KdN41* gene improved tobacco drought resistance but failed to improve the resistance of tobacco plants to salt and heat stress. This indicates that this gene could play an indirect role in clearing harmful substances induced by stress. The lack of response of this gene during salt and heat stress indicates that systematic control of the specific response gene is crucial for the perception of certain environmental stresses.

The reduced leaf size of the *KdN41*-overexpressing plants is suggestive of several roles for this gene. According to the data (Supplementary Table S3), the leaves of *KdN41*-overexpressing plants were much shorter and narrower than those of WT and NC plants. These reduced leaf areas provide benefits for drought resistance. For example, lower work load for various physiologic traits such as leaf hydraulic conductance, photosynthesis, and nutrition consumption might be achieved^18^. In addition, lower membrane damage, lower levels of oxidant toxin levels and osmotic adjustment substances would be achieved^19^. The latter was consistent with the physiological parameters we observed (Fig. 6). In addition, tissue-specific expression of *KdN41* gene in vascular tissue under drought stress (Fig. 3) reflects its possible role in communicating with other regulators in the leaf hydraulic conductance photosynthesis and nutrition consumption processes. The leaf vein is an essential structure in nutrition, water, and hormone transportation during the plant life span, and supports photosynthesis^20^. The leaf hydraulic conductance machinery can manipulate water transport and keep the stomata open to allow photosynthesis^21^. Previous study also confirmed that reduced cell size rather than cell number will determine the plasticity in vein and stomata density, which adjusts leaf transpiration in dealing with changing environment conditions^22^. The *KdN41*-overexpressing plants showed an obvious reduced decrease in photosynthetic rate compared to the WT (Supplementary Fig. S12b,e and h) and NC (Supplementary Fig. S12c,f and i) plants, especially under medium and severe drought stress (Supplementary Fig. S12d–f and g–i).

Thus, the reduced leaf size of *KdN41*-overexpressing tobacco might also serve as a dynamic leaf transpiration system to adapt to drought stress condition. In summary, the reduced size of leaves in *KdN41*-overexpressing tobacco may be an adaptive in response to drought stress.

Since the first report on the function of DREB transcription factor^23^, dwarfing plants has been a classic way to enhance stress tolerance^24^. However, some studies have confirmed improved stress tolerance in *DREB1B*-overexpressing plants without dwarfing, suggesting that ABA signaling is an independent stress response mechanism^25^. In this report, differences in ABA concentration between different kinds of transgenic plants and WT were much smaller than other physiological parameters, which might suggest that *KdN41*-overexpressing tobacco possess different methods of improving drought resistance.

During light to severe drought stress, the *KdN41*-overexpressing tobacco leaves showed high to low antioxidant enzyme activities compared to WT and NC plants (Fig. 6), indicative of hypersensitivity in detoxifying the oxidant substances. During drought stress, the *KdN41*-overexpressing plants continued to grow taller, but the leaf length and width were much lower than WT and NC plants (Supplementary Table S3). This could also be explained by the lower expression levels of auxin-related genes and higher expression of the *ABA responsive element binding factor* (*ABF*) gene, which endows *KdN41*-overexpressing plants with a slower growth rate than WT and NC plants under continuous drought stress (Supplementary Fig. S13). Therefore, the *KdN41* gene in *KdN41*-overexpressing plants may be involved in balancing growth and drought stress resistance, and the low ratio of growth to drought stress resistance in *KdN41*-overexpressing plants is a result of “fine-tuning” of drought stress resistance.

The main approach in modern crop breeding is to cultivate a single species with multiple key agronomic traits (seed quality, biomass production, and pest and disease resistance) to cope with rapidly changing environmental conditions. For this purpose, low-input, space, and resource conservation, as well as biodiverse agriculture or horticulture, is essential^26^. *K. daigremontiana* is an ideal plant with rapid propagation and various environmental condition adaptation traits in agriculture and horticulture usage.

The *KdN41* gene is an interesting example that reflects precise control between growth rate and drought stress resistance. In this study, the 35 S promoter was used to drive *KdN41* gene expression in various tissues. *KdN41* overexpression was found to confer improved drought resistance, but it could also decrease biomass production due to reduced leaf size in transgenic tobacco. However, an inducible or tissue-specific promoter to control *KdN41* gene expression could be used to regulate expression of this gene in specific situations. One potential approach is to apply the RD29A promoter (a specific drought response promoter) to regulate *KdN41* gene expression. During water shortages in the drought season, this gene may function in stressed crop tissues to enhance the survival rate. When the water availability returns to normal, the crop will provide better biomass production compared to non-breeding cultivars. Considering the early flowering phenomenon in *KdN41*-overexpressing tobacco, this could be beneficial for breeding based on the crop flowering time. Generating a crop with a controllable flowering time in a specific season would address the late flowering problem in agriculture and horticulture production. Future studies on other gene candidates for crop breeding may be possible by exploring the *KdN41* gene network.

## Methods

### Plant materials and growth conditions

Tobacco and *K. daigremontiana* were grown in substrate (peat:perlite = 3:1) under a 16/8-h light (250 μmol m^−2^ s^−1^) cycle at 25 °C at 50–70% relative humidity in a growth room.

### RACE assay, Genome-walking assay and bioinformatics analysis of *KdN41* gene

DNA and RNA extractions from *K. daigremontiana* were performed as described previously^27,28^. Based on a partial cDNA sequence, TaKaRa 5′-Full-RACE (TaKaRa, Japan) and TaKaRa 3′-Full-RACE (TaKaRa, Japan) kits were used to amplify the 5′- and 3′-ends of the full length *KdN41* cDNA sequence with cDNA template. According to a partial gDNA sequence of *KdN41* gene, TaKaRa Genome Walking Kit (TaKaRa, Japan) was employed to amplify 5′ flanking sequence containing promoter. The *KdN41* gene ORF was predicted using the ORF Finder program (http://www.ncbi.nlm.nih.gov/gorf/gorf.html), and *cis*-acting elements in the promoter were predicted using the Plant Cis-Acting Regulatory Element (PlantCare; http://bioinformatics.psb.ugent.be/webtools/plantcare/html/) database. Conserved domains were predicted using the NCBI Conserved Domain Search program (http://www.ncbi.nlm.nih.gov/Structure/cdd/wrpsb.cgi), and CDS-encoding protein characteristics were predicted using the ProtParam (http://web.expasy.org/protparam/), iPSORT (http://ipsort.hgc.jp/), and PSIPRED (http://bioinf.cs.ucl.ac.uk/psipred/) programs.

### Hormone treatments in *K. daigremontiana*

Three-month-old WT *K. daigremontiana* plants were divided into five groups. All groups were treated using the foliage spray method, as follows: 500 mL H_2_O (CK), 500 mL of 100 μM ABA (product number: A1049, Sigma, USA), 500 mL of 100 μM SA (product number: S7401, Sigma, USA), 500 mL of 100 μM GA_3_ (product number: G7645, Sigma, USA), and 500 mL of 100 μM MeJa (product number: 392707 ALDRICH, USA) respectively. RNA was extracted from the leaves of three individual plants (each representing a biological replicate) from each group after 2, 4, 6, and 10 h. cDNA synthesis and real-time PCR analyses were performed using the TransStart® Tip Green qPCR SuperMix (TransGen Biotech, Beijing), following the manufacturer’s instructions, and an ABI Step One PCR instrument (Applied Biosystems). The results were calculated using the 2^−ΔΔCt^ method^29^. The assay for each gene used three biological replicates as templates (For each biological replicate, three technical replicates were used. Thus, a total of nine reactions were run.) The partial cDNA sequence of the *KdActin* gene was used as a reference gene. Because this gene indicated stable expression level among different plant organs including leaf, stem and root in previous study. Leaf of CK was treated as a reference tissue.

### Vector construction and tobacco transformation

The complete *KdN41* ORF cDNA sequence was introduced into the pBIN438 plasmid driven by the enhanced CaMV35S promoter as an overexpression construct (35*S::N41*); A tissue expression construct of the *KdN41* gene (*Promoter*_*KdN41*_*::GUS*) was designed by using its 197-bp promoter sequence to replace the CaMV35s promoter in the pBI121 plasmid, to drive expression of the *GUS* gene. The empty plasmids pBIN438 (35*S::None*) and pBI121 (35*S::GUS*) were used as a NC and PC in further experiments. YFP was employed as a reporter. Recombinant pGTVII plasmids containing 35*S::N41:*:*YFP* and 35*S::YFP* (as control) were constructed respectively. All plasmids mentioned above were transformed into the *Agrobacterium tumefaciens* strain LBA4404.

The tobacco plants were infected by *A. tumefaciens* strain LBA4404 with the constructs mentioned above through previous method^30^. Confirmation of positive transformation in tobacco T0 generation of each construct was performed by PCR amplification of the *KdN41* gene ORF, *KdN41* promoter, partial *NPTII* respectively (Supplementary Fig. S14–S17). Three primary transformed tobacco plants of each construct were selected randomly to provide T1 progeny. Seeds of primary transgenic plants were grown on Murashige and Skoog (MS) medium with a 50−mg L^−1^ kanamycin selection pressure for three weeks (WT seeds were grown on MS medium) (Supplementary Table S4).

### Transgenic tobacco line stress treatments

#### Drought stress treatment

T1 seedlings from three individual lines of each construct were used, and for each line, three seedlings were employed (nine seedlings in total). The combination of *Promoter*_*KdN41*_*::GUS*, PC, and WT (for *KdN41* tissue expression) and the combination of OE, NC, and WT (for *KdN41* function identification) were grown in MS liquid medium with 20% PEG6000 for 10 h as a drought stress treatment (controls were grown in liquid MS medium) at 25 °C under a 16/8-h light (250 μmol m^−2^ s^−1^) cycle at 50–70% relative humidity (Supplementary Table S5).

Three-month-old T1 generation plants from three individual lines of each construct were used (three biological replicates for each individual line, and nine plants per construct). *KdN41* OE, WT, and NC tobacco plants grown in peat substrate were watered to pot capacity and allowed to dry gradually. After the first sign of wilting was observed, soil water content was measured using a Delta T wet sensor (Delta-T Devices Ltd., UK). Three weeks after soil drying, LD was reached at 8–10% (8.34% average) soil water content; four weeks after soil drying, MD was reached at 3–5% (3.42% average) soil water content; five weeks after soil drying, severe SD was reached below 1% (0.21% average) soil water content. Each kind of transgenic plant and WT contained a non-treatment group (NT) that remained well-watered as a control (Supplementary Table S5).

#### Salt stress treatment

T1 seedlings from three individual lines of each construct were used, and for each line, fifteen seedlings were employed (forty-five seedlings per construct). The combination of *Promoter*_*KdN41*_*::GUS*, PC, and WT (for *KdN41* tissue expression) and the combination of OE, NC, and WT (for *KdN41* function identification) were treated as follows (Supplementary Table S5): grown on solid MS medium containing 400 mM NaCl at 25 °C under a 16/8-h light (250 μmol m^−2^ s^−1^) cycle at 50–70% relative humidity for 7 d for salt stress treatment (controls were grown on MS medium).

#### Heat stress treatment

T1 seedlings from three individual lines of each construct were used, and for each line, fifteen seedlings were employed (forty-five seedlings per construct). The combination of *Promoter*_*KdN41*_*::GUS*, PC, and WT (for *KdN41* tissue expression) and the combination of OE, NC, and WT (for *KdN41* function identification) were treated as follows (Supplementary Table S5): grown on MS medium at 50 °C for 7 h in a growth chamber for heat stress treatment (controls were grown on MS medium at 25 °C).

### Histochemical staining assay

For GUS protein expression localization, tobacco leaves were stained as described previously^31^. H_2_O_2_ localization in tobacco leaves was detected using the DAB and NBT staining methods^32^. Images are representative of >10 observed samples stained in three independent experiments for each stress treatment.

### YFP localization assay

One-month old tobacco leaves were injected with the LBA4404 strain containing one of the plasmids mentioned above, as described previously^33^. After 48 h, microscopic observations for YFP or KdN41-YFP fusion protein were performed using a Confocal Laser Scanning Platform Leica TCS SP8 (Leica, Germany).

### Physiological traits and gene expression quantification

The photosynthetic activities of the three uppermost fully developed leaves were measured at three individual stress stages using a Li-6400 (LI-COR, USA). POD and CAT activities of leaves were measured following a previously described method^34^. EL of 0.1 g of fresh leaves from three individual stress stages was measured using a previously described method^35^. Proline, MDA, H_2_O_2_ and O_2_^−^ contents were measured following a previously described method^32^. Total RNA was extracted from 0.1 g of tobacco leaves using the Eastep® Super Total RNA Extraction Kit (Promega, Shanghai) and cDNA was synthesized using the GoScript™ Reverse Transcription System (Promega, Shanghai) and real-time PCR was performed using the TransStart® Tip Green qPCR SuperMix (TransGen Biotech, Beijing), following the manufacturer instructions, using an ABI Step One PCR instrument (Applied Biosystems). The results were calculated using the 2^−ΔΔCt^ method^29^. The reference gene used for Quantitative Real-time PCR was *NtEF1α*, which is a housekeeping gene (GenBank accession no. D63396). WT tobacco leaf of NT was chosen as a reference tissue. Three individual lines (three plants for each line as technical replicates, totally nine plants) of OE, WT, and NC were used as biological replicates.

### Hormone detection in transgenic tobacco plants

Raw hormone mixtures were extracted from 0.1 g of tissue from the uppermost fully developed leaf with 1 mL of pH 7.0 PBS. ABA concentrations were measured using a colorimetric ELISA kit containing plates pre-coated with antibody specific to ABA. (Winter Song Boye Biotechnology Co. Ltd., Beijing). Samples were diluted six-fold prior to the tests. The standard curve was generated based on a series of known ABA standard sample reactions to 180, 120, 60, 30, 15, and 0 ng mL^−1^. Three individual lines from each transgenic plant and WT were used. Three biological replicates of each individual line of each kind of transgenic plant were used.

### Bioinformatic analysis of SSH library

A SSH library from the leaves of *K. daigremontiana* induced by drought stress was obtained as previous described^16^. Different expressed genes (DEGs) as ESTs between normal watering and drought treatment samples were further annotated by Nr, Nt, Swiss-prot, and KEGG, and classified by COG and GO.

### Statistical analyses

ANOVA and mean comparisons were performed using SPSS version 20.0 software. The value of 2^−ΔΔCT^ was also proceeded with SPSS version 20.0 software. Error bars represent standard deviation. *And different letters indicate statistically significant differences at *p* < 0.05 based on Duncan’s multiple range test. Figures were created using SciDavis 0.2.4.

### Primers

Primers were listed in supplementary materials (Supplementary Table S6).

## Electronic supplementary material

Supplementary Dataset 1
