# Supplementary material for: A novel gene of Kalanchoe daigremontiana confers plant drought resistance
Source: Sci Rep. 2018 Feb 7;8:2547. doi: 10.1038/s41598-018-20687-5 (PMC5803263; doi:10.1038/s41598-018-20687-5)
Supplement: Supplementary file 1 — Supplementary Dataset 1 [file 41598_2018_20687_MOESM1_ESM.doc]

**A novel gene of *Kalanchoe daigremontiana* regulates growth and drought resistance**

Running title: **A novel gene impacting growth and drought tolerance**

**Li Wang^1*^, Chen Zhu^2*^, Lin Jin,^3^Aihua Xiao^1^, Jie Duan^4^, Luyi Ma^1＋^**

**^＋^Correspondence:** Luyi Ma, maluyi@bjfu.edu.cn

**^*^**These authors contributed equally to this work.

**
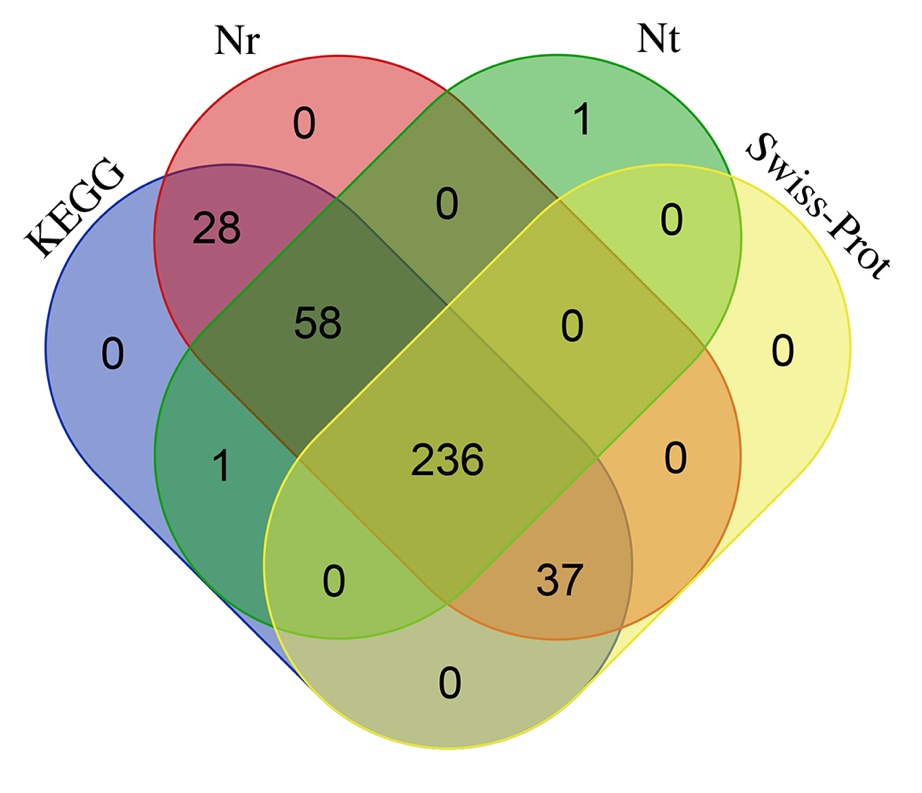
**

**Supplementary Fig. S1 Venn diagram showing the numbers of gene been annotated resulted from drought stress *K. daigremontiana*.**

Nr, non-redundant protein database; Nt, non-redundant nucleotide database; Swiss-Prot, Swiss-Prot protein database; KEGG, Kyoto encyclopedia of genes and genomes database.

**
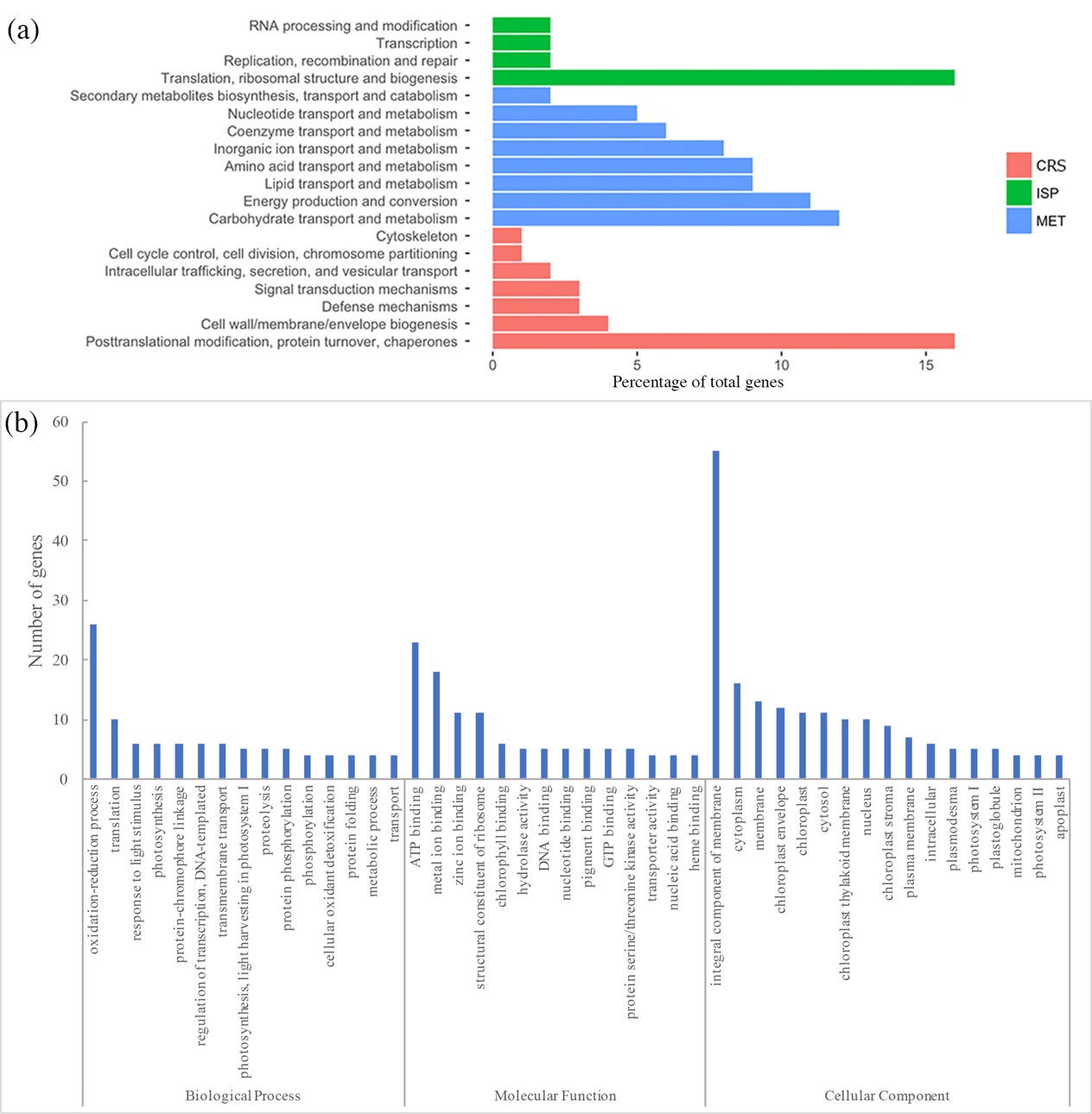
**

**Supplementary Fig. S2 COG (a) and GO (b) Function Classification of all annotated ESTs resulted from drought stress in *K. daigremontiana***

**CPS, Cell Processes and Signaling; ISP, Information Storage and processing; MET, Metabolism.**


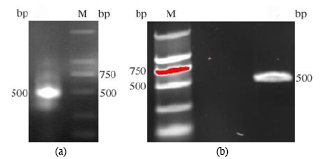


**Supplementary Fig. S3 PCR results of 3’ RACE (A) and 5’ RACE (B).**


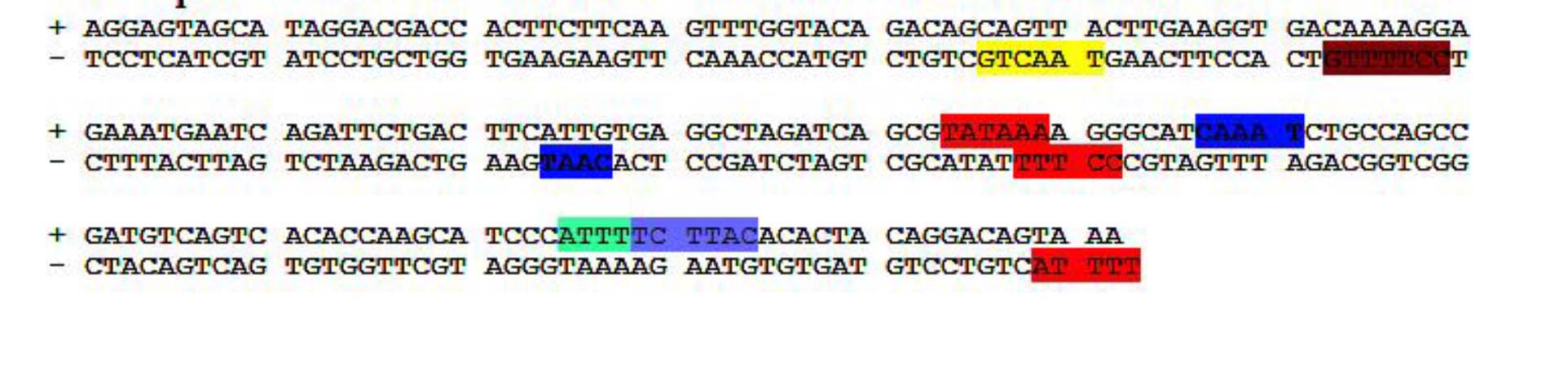


**Supplementary Fig. S4 Sequence analysis of *KdN41* gene promoter.** Blue: CAAT-box; Yellow: MBS (**M**YB TF **B**inding **S**ite); Brown: P-box; Red: TATA-box; Green: TC-rich repeats; Light blue: TCT: motif


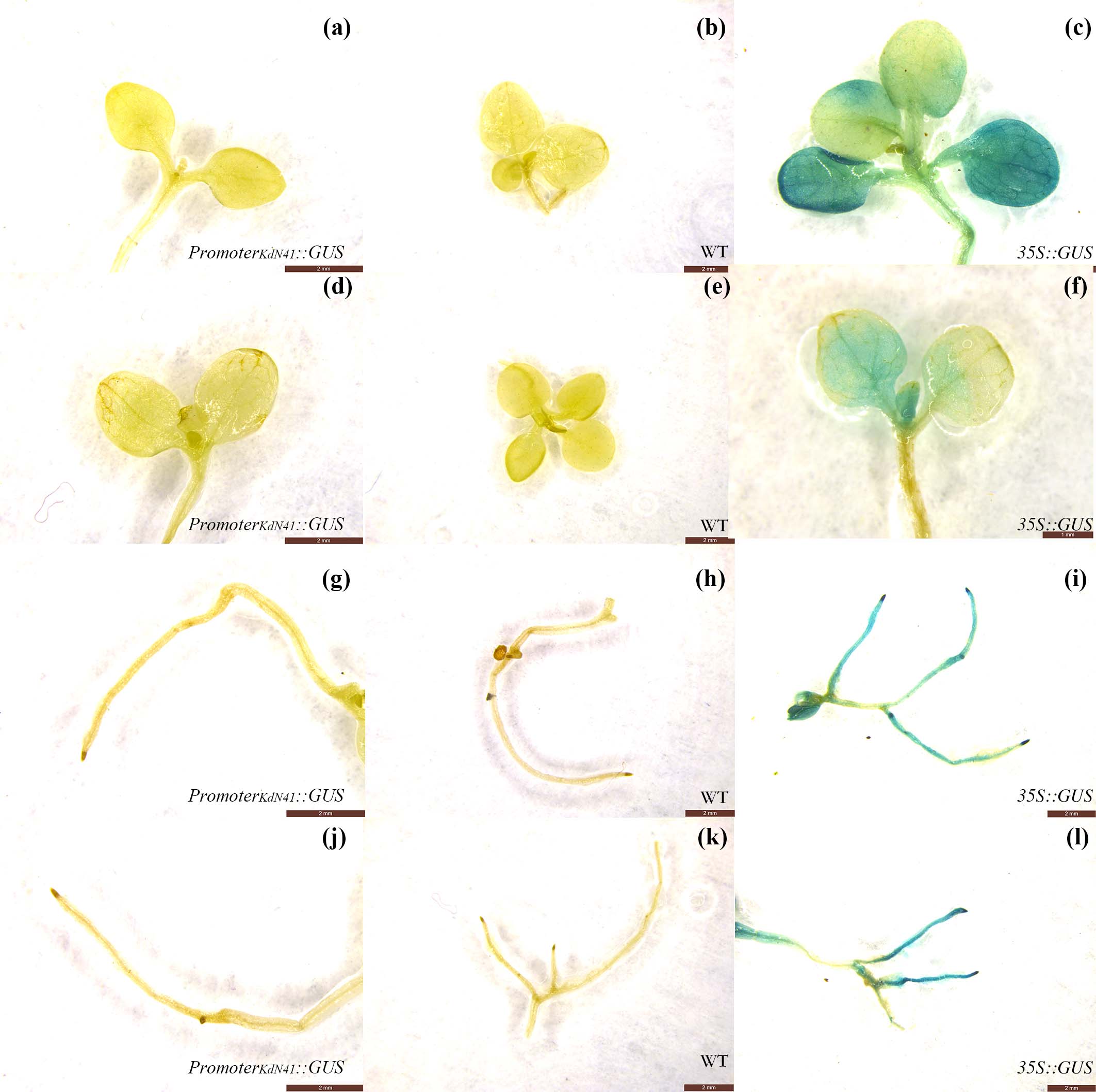


**Supplementary Fig. S5 *KdN41* GUS staining analyzes of *Promoter_KdN41_::GUS*, wild type (WT), and *35S::GUS* (PC)** **plants after salt stress.** a-c: leaf staining results with no salt treatment; d-f: leaf staining results under salt treatment; g-i: root staining results with no salt treatment; j-l: root staining results under salt treatment.


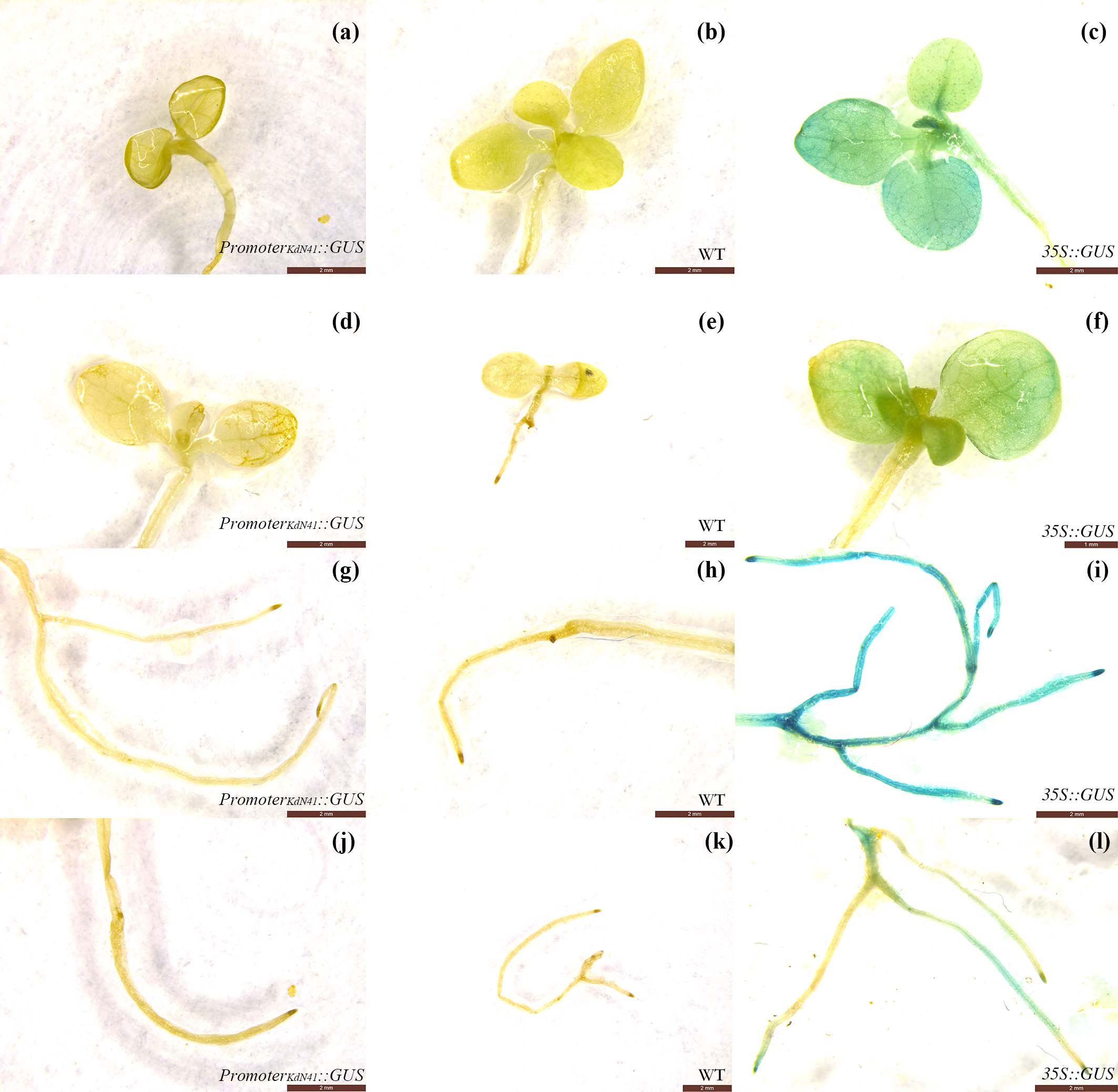


**Supplementary Fig. S6 *KdN41* GUS staining analyzes of *Promoter_KdN41_::GUS*, wild type (WT), and *35S::GUS* (PC)** **plants after heat stress.** a-c: leaf staining results with no heat treatment; d-f: leaf staining results under heat treatment; g-i: root staining results with no heat treatment; j-l: root staining results under heat treatment.


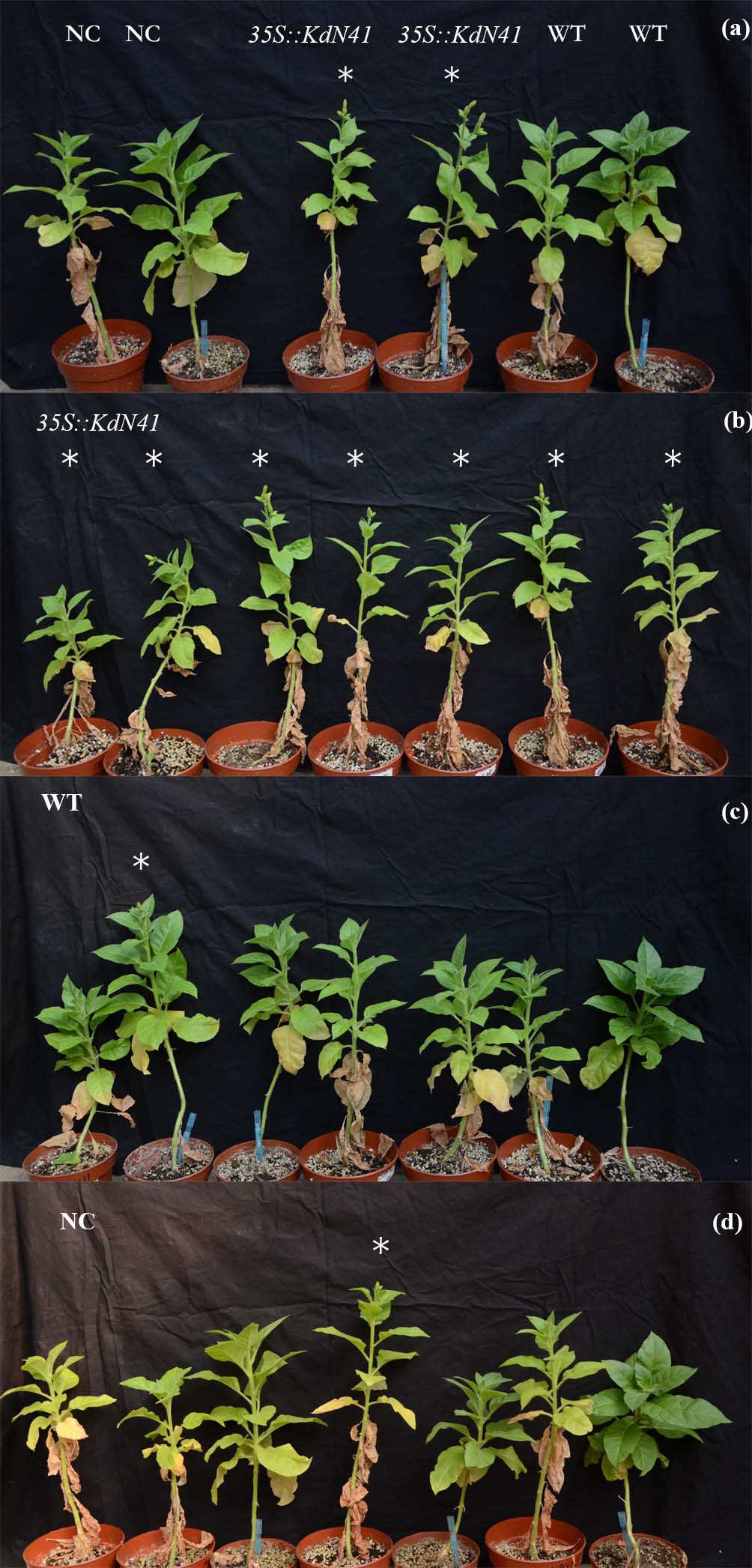


**Supplementary Fig. S7 Flowering comparison among *35S::N41* (OE), WT (wild type) and NC (negative control) tobacco plants re-watered after severe drought stress.** a: Parallel comparison between OE, WT, and NC plants at the same time; b: OE flowering results; c: WT flowering results; d: NC flowering results;* indicates flowering phenotype. All the pictures were taken at the last day of week 21 since planting.


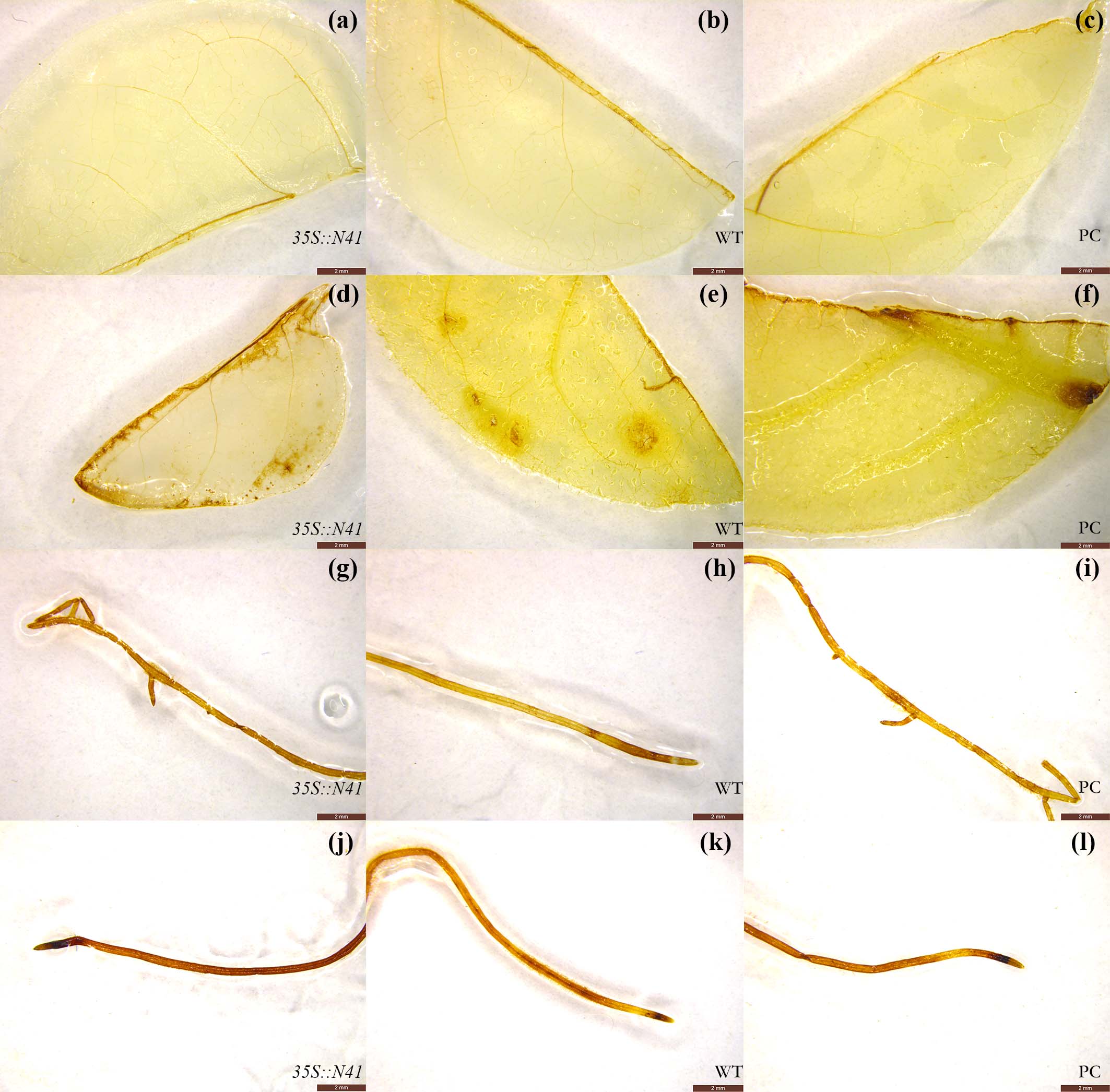


**Supplementary Fig. S8 DAB staining analyzes of *Promoter_KdN41_::GUS*, wild type (WT), and PC (positive control, *35S::GUS*)** **plants after drought stress using 20% PEG.** a-c: leaf staining results with no drought treatment; d-f: leaf staining results under drought treatment; g-i: root staining results with no drought treatment; j-l: root staining results under drought treatment; Black arrow points out the GUS staining area in leaf vein of *Promoter_KdN41_::GUS* plants.


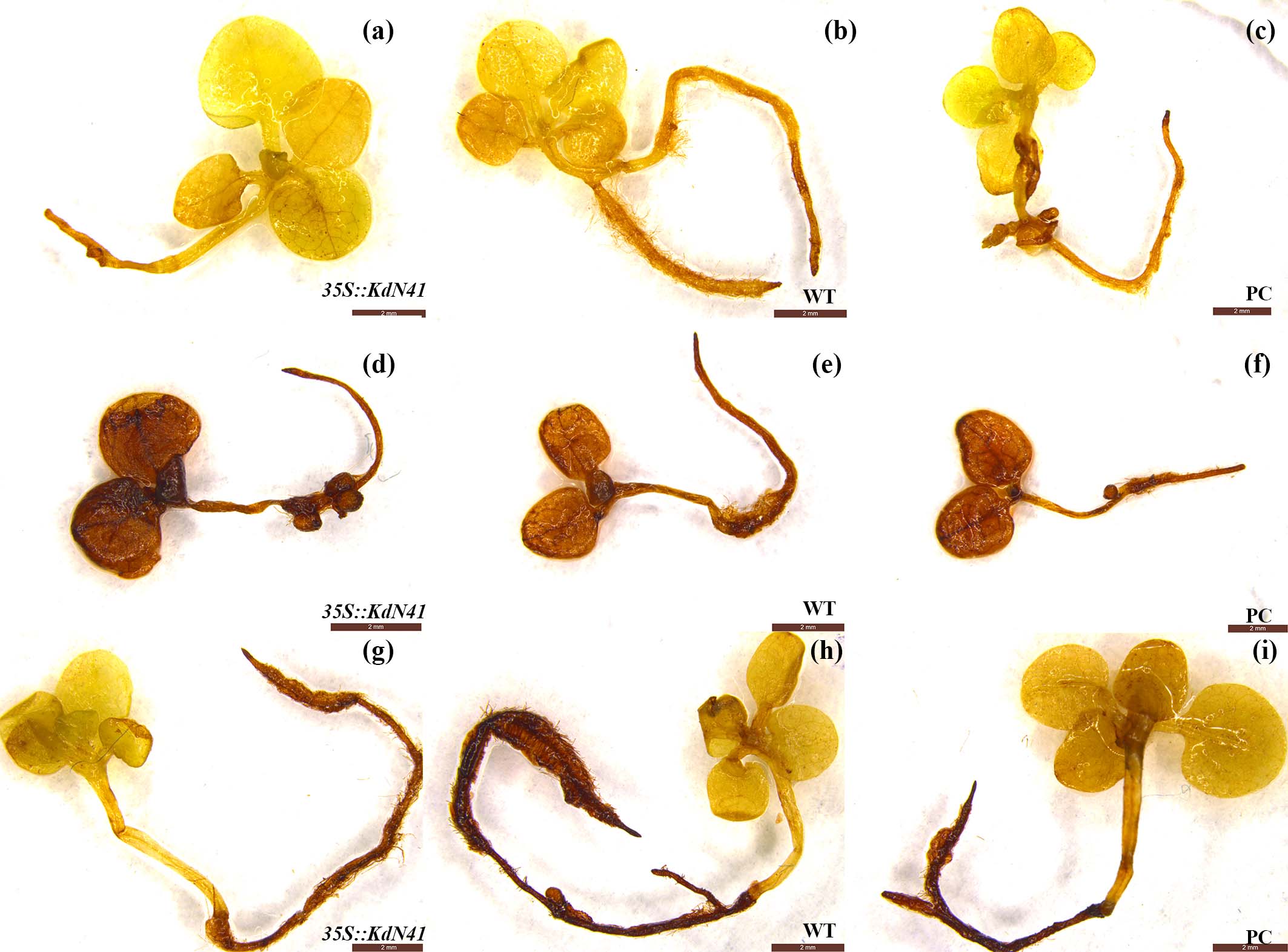


**Supplementary Fig. S9 DAB staining analyzes after salt and heat treatment.** a-c: *35S::N41* (OE), WT (wild type), and PC (positive control) plants staining results with no treatment; d-f: OE, WT, and PC plant staining results after salt stress; g-i: OE, WT, and PC plant staining results after heat stress.


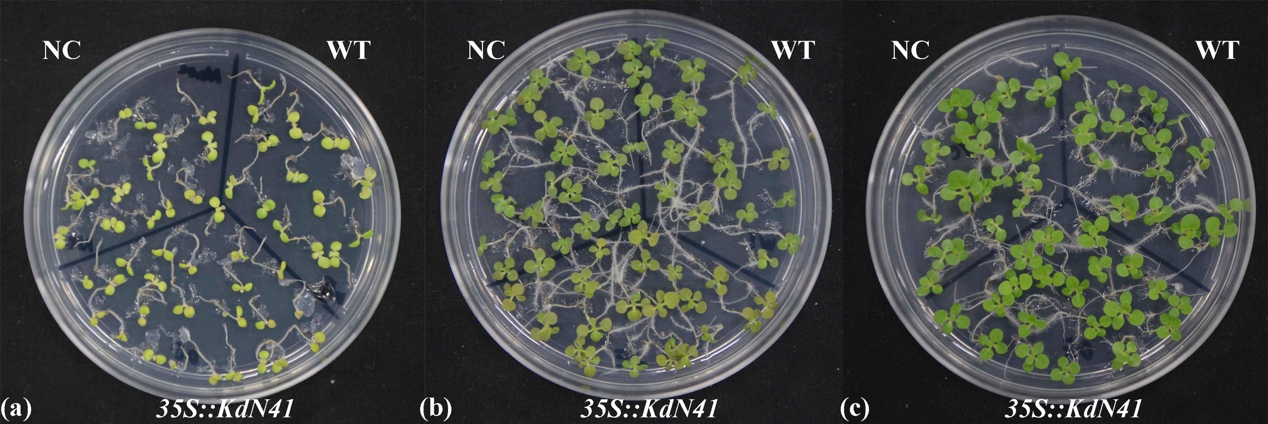


**Supplementary Fig. S10 *35S::N41* (OE), wild type (WT) and NC (negative control) tobacco seedlings showed no difference in response to salt and heat stress.** a: OE, WT, and NC plants under salt stress; b: OE, WT, and NC plants under heat stress; c: OE, WT, and NC plants with no stress treatment.


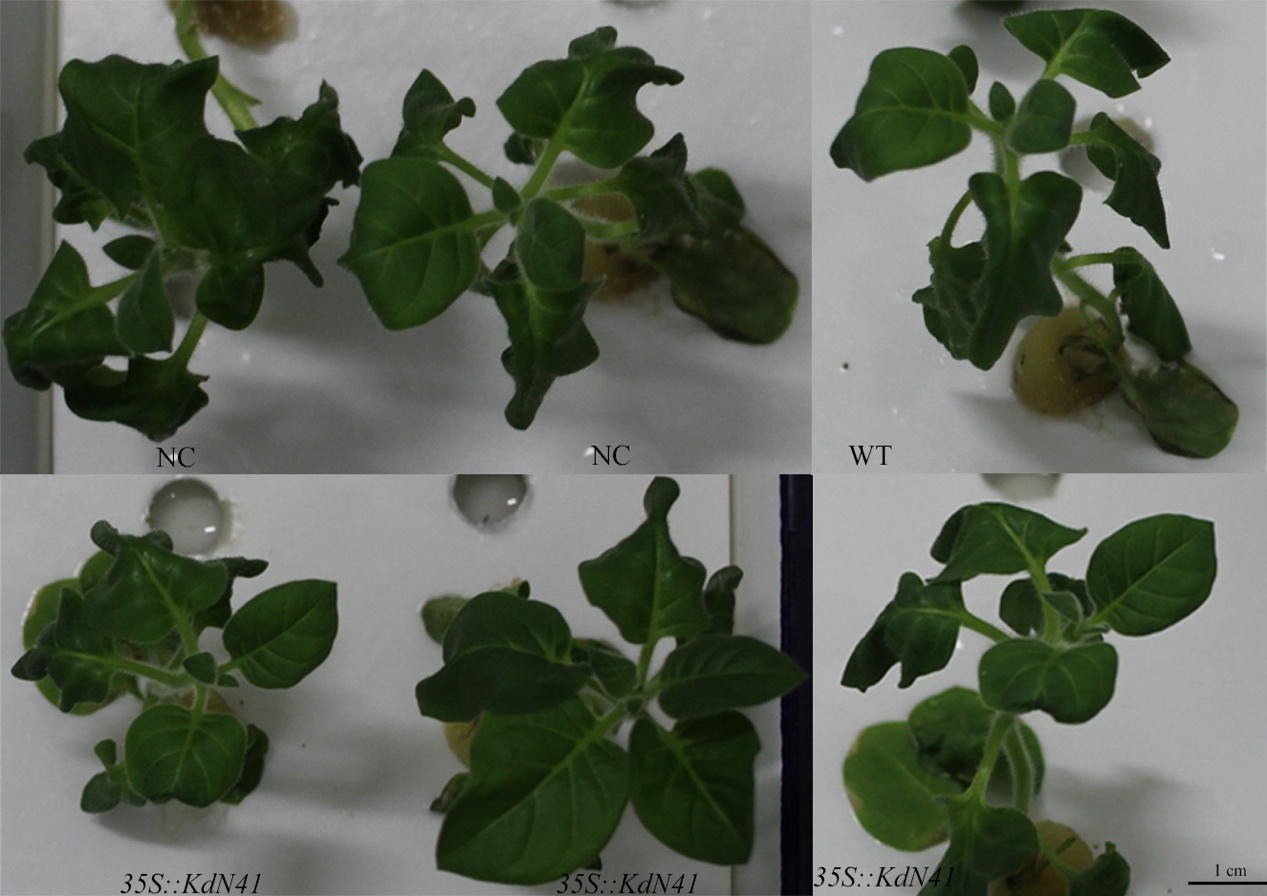


**Supplementary Fig. S11 *35S::N41* (OE) tobacco seedlings indicated less wilting phenotype than wild type (WT) and NC (*35S::None*) in response to PEG drought stress.**


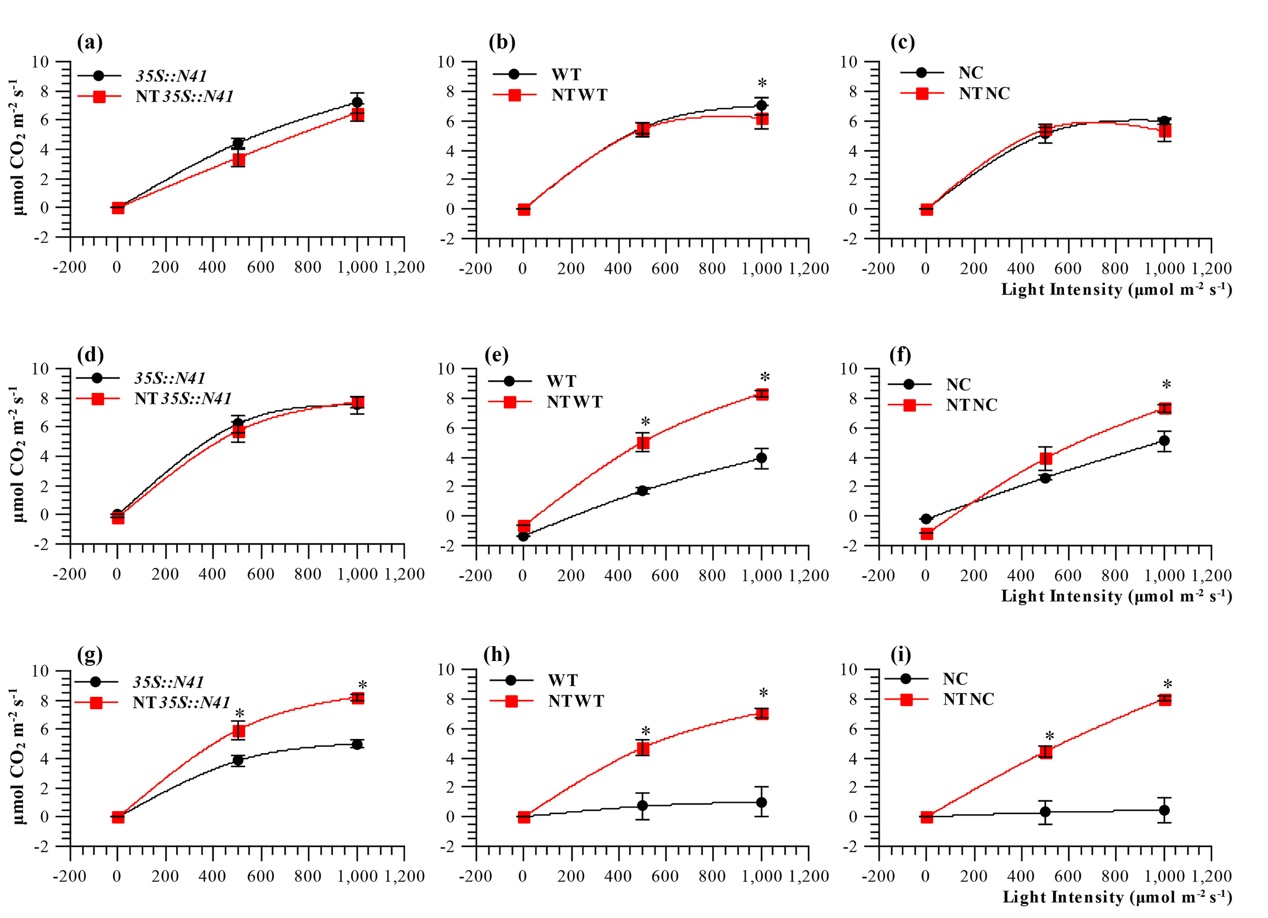
 **Supplementary Fig. S12 Photosynthetic rates at different light intensities in *35S::N41* (OE), wild type (WT) and NC (negative control) tobacco plants in response to increasing drought stress.** The red lines represent NT (non-treatment); a-c: black lines represent plants under light drought stress; d-f: black lines stand for plants under medium drought stress; g-i: black lines stand for plants under severe drought stress. Photosynthetic rate was assessed under 0, 500, and 1000 μmol m^-2^ s^-1^. * indicates a significant difference within drought treatment compared with NT (Duncan’s multiple range test P<0.05).

**
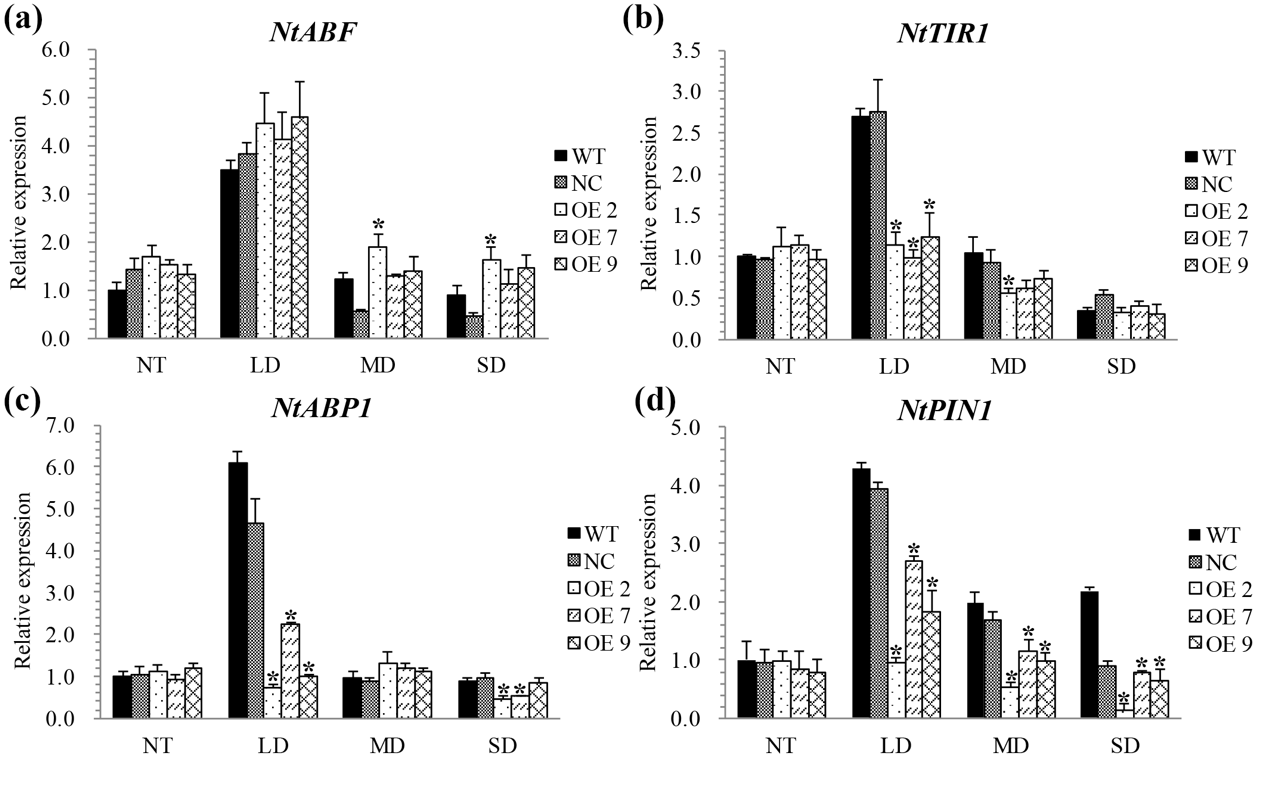
 Supplementary Fig. S13 Relative expressions of *NtABF*, *NtTIR1*, *NtABP1*, and *NtPIN*, and genes in *35S::N41* (OE), wild type (WT) and NC (negative control) tobacco plants in response to increasing levels of drought stress, NT (non-treatment), LD (light drought), MD (medium drought), and SD (severe drought).** * indicated significant difference compared to WT (Duncan’s multiple range test *p* < 0.05). The expression level of WT group was treated as reference and calculated as 1.


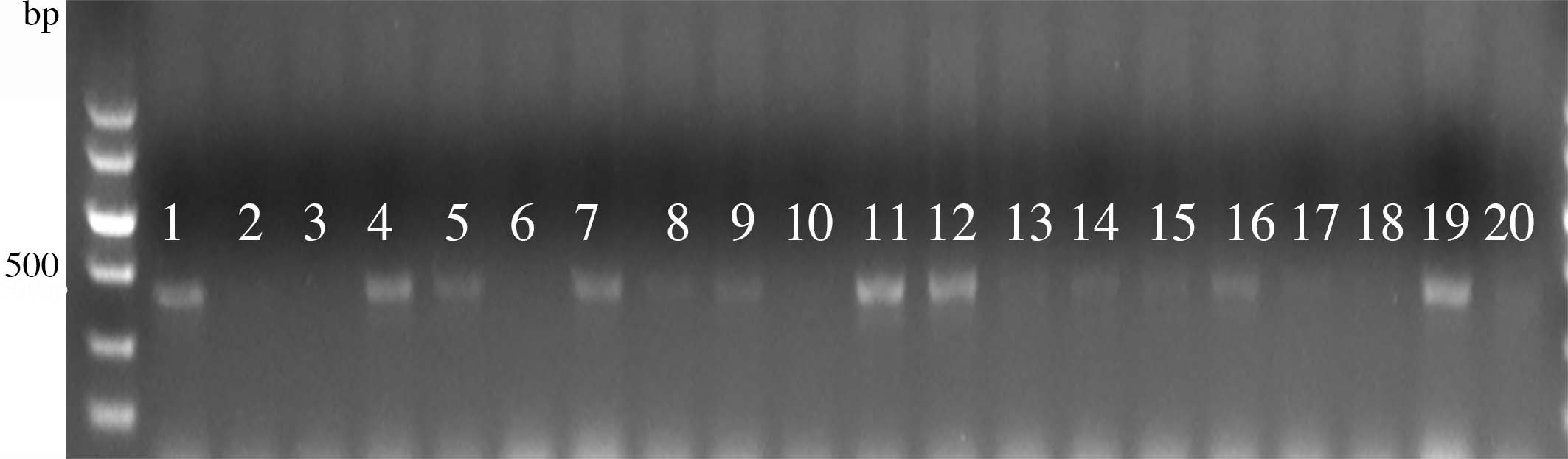


**Supplementary Fig. S14 OE (*35S::N41*) positive line detection of T0 tobacco generation through PCR method.** 1, 4, 5, 7, 8, 9, 11, 12, 14, 15, 16, 17, and 19 were confirmed as OE positive lines; 2, 3, 6, 10, 13, 18, and 20 were confirmed as false positive lines.


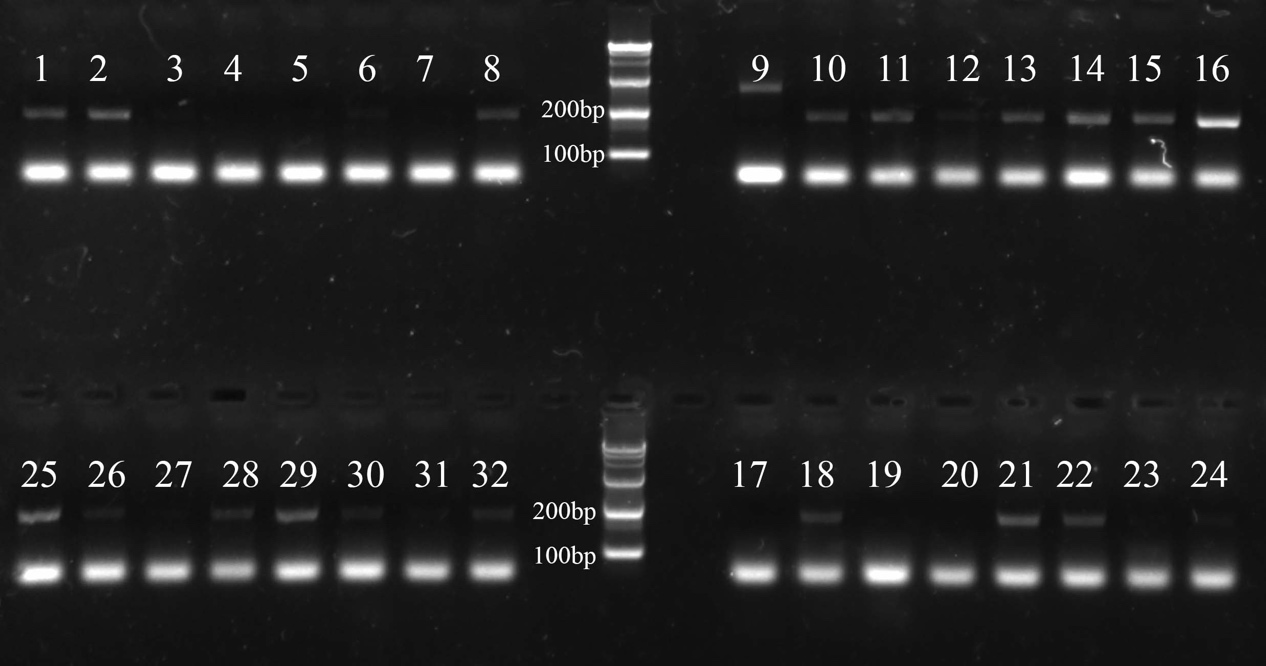


**Supplementary Fig. S15*Promoter_KdN41_::GUS* positive line detection of T0 tobacco generation through PCR method.** 1, 2, 10, 11, 13, 14, 15, 16, 18, 21, 22, 25, 26, 28, 29, 30, and 32 were confirmed as positive lines, and the rest were confirmed as false positive lines.


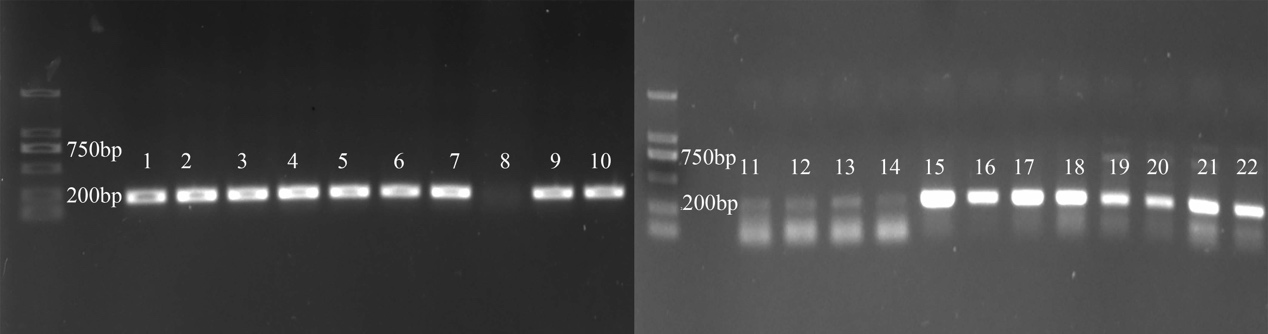


**Supplementary Fig. S16 *35S::GUS* (PC) positive line detection of T0 tobacco generation through PCR method.** *NTPII* gene was amplified. 1, 2, 3, 4, 5, 6, 7, 9, 10, 11, 12, 13, 14, 15, 16, 17, 18, 19, 20, 21, and 22 were confirmed as positive lines, 8 was confirmed as false positive lines.


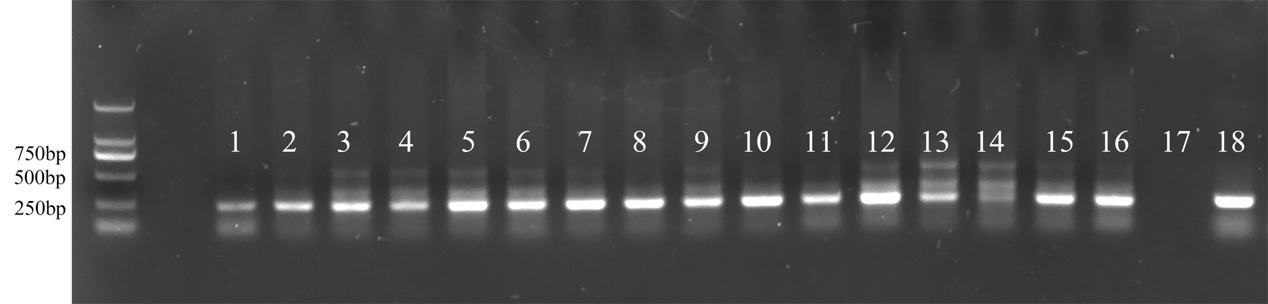


**Supplementary Fig. S17 *35S::None* (NC) positive line detection of T0 tobacco generation through PCR method.** *NTPII* gene was amplified. 1, 2, 3, 4, 5, 6, 7, 8, 9, 10, 11, 12, 13, 14, 15, 16, and 18 were confirmed as positive lines, 8 was confirmed as false positive lines.

**Supplementary Table S1. Genes of oxidation-reduction process** in SSH library

| Query name | Annotation | GenBank accession |
| --- | --- | --- |
| 124_M13-47_F05_1103070983J | 2-Cys peroxiredoxin | JK317328.1 |
| 194_M13-47_D04_1103074043J | protochlorophyllide oxidoreductase | JK340149.1 |
| 221_M13-47_E02_1103075718J | L-ascorbate peroxidase, cytosolic | JK340156.1 |
| 277_M13-47_A06_1103074068J | Dihydrolipoyl dehydrogenase | JK340164.1 |
| 365_M13-47_A05_1103074099J | glyceraldehyde-3-phosphate dehydrogenase, NADP binding | JK340185.1 |
| 462_M13-47_H05_1103075722J | linoleate 13S-lipoxygenase | JK340199.1 |
| 523_M13-47_F04_1103075725J | NADH dehydrogenase | JK340229.1 |
| 903_M13-47_C09_1103077726J | peroxidase | JK340311.1 |
| 1040_M13-47_E03_1103077656J | histidinol chloroplastic | JK317256.1 |
| 1129_M13-47_G06_1103077698J | ferredoxin--NADP | JK317279.1 |
| 1134_M13-47_D10_1103077700J | 2-alkenal reductase (NADP(+)-dependent) | JK317281.1 |
| 1232_M13-47_A05_1104060282J | cytochrome | JK317324.1 |
| 1266_M13-47_C06_1104060292J | thiamine thiazole synthase | JK317333.1 |
| 1314_M13-47_D08_1104060309J | zeaxanthin epoxidase | JK340000.1 |
| 1409_M13-47_B03_1104060353J | magnesium-protoporphyrin monomethyl ester | JK340041.1 |
| 1521_M13-47_F03_1104061310J | glutamyl-tRNA reductase-binding | JK340085.1 |
| 1538_M13-47_H04_1104061320J | DSBA oxidoreductase | JK340094.1 |
| LA11001-66.CleanEST.seq.Contig7 | 1,2-dihydroxy-3-keto-5-methylthiopentene dioxygenase 2 | JK317261.1 |
| LA11001-66.CleanEST.seq.Contig13 | glyceraldehyde-3-phosphate, NADP binding | JK317285.1 |
| LA11001-66.CleanEST.seq.Contig24 | protoporphyrinogen oxidase | JK340033.1 |
| LA11001-66.CleanEST.seq.Contig27 | NADPH-ferrihemoprotein reductase | JK340058.1 |
| LA11001-66.CleanEST.seq.Contig30 | 2-alkenal reductase (NAD(P) (+)) | JK340068.1 |
| LA11001-66.CleanEST.seq.Contig35 | Ferredoxin-dependent glutamate | JK340079.1 |
| LA11001-66.CleanEST.seq.Contig37 | CAT2; catalase | JK340140.1 |
| LA11001-66.CleanEST.seq.Contig45 | oxygen-evolving enhancer | JK317259.1 |
| LA11001-66.CleanEST.seq.Contig46 | pyrophosphate-energized vacuolar membrane proton pump | JK340197.1 |
| LA11001-66.CleanEST.seq.Contig47 | CAT2; catalase | JK317334.1 |

**Supplementary Table S2**. The proportion of flower buds of OE, WT and NC tobacco exposed to severe drought stress and re-watering determined 20 to 24 weeks after planting.

| Plant Type  (total bud numbers) | Week 20 | Week 21 | Week 22 | Week 24 |
| --- | --- | --- | --- | --- |
| OE (24) | 50.00% | 100.00% | 100.00% | 100.00% |
| WT (29) | 0.00% | 10.34% | 20.69% | 100.00% |
| NC (31) | 0.00% | 9.68% | 22.58% | 100.00% |

OE: *KdN41* overexpression plants; WT: wild type plants; NC: negative control plants (transformed with empty vector). For each kind of transgenic plant and WT, totally 7 individual plants were employed. Bud numbers was counted and gathered from all the 7 plants.

**Supplementary Table S3** Tobacco plant growth during increased drought stress.

| Drought stress | Leaf length(cm) | | | Leaf width(cm) | | | Plant height(cm) | | |
| --- | --- | --- | --- | --- | --- | --- | --- | --- | --- |
|  | OE | WT | NC | OE | WT | NC | OE | WT | NC |
| NT | 8.00±0.67 b | 10.77±0.78 a | 9.03±1.02 ab | 4.32±0.20 b | 5.61±0.33 a | 5.23±0.52 a | 23.4±1.17 a | 22.08±1.0 9 a | 22.96±1.92 a |
| LD | 8.99±1.90 ab | 11.02±0.96 a | 10.11±1.30 a | 4.07±0.60 c | 5.91±1.19 ab | 5.60±1.65 ab | 19.81±2.20 b | 19.42±1.59 b | 18.10±1.97 b |
| MD | 8.63±0.38 b | 9.93±0.38 a | 9.63±0.24 ab | 4.70±0.24 b | 5.93±0.64 a | 5.23±0.63 ab | 22.75±1.66 ab | 20.33±1.71 ab | 19.50±1.73 b |
| SD | 7.67±1.33 b | 8.97±0.89 ab | 7.80±1.59 b | 3.67±0.23 c | 4.43±0.60 bc | 4.2±0.20 bc | 24.33±1.53 a | 19.63±1.12 b | 20.00±1.00 b |

Different letters indicated significant difference between each two groups of one parameter (leaf length, leaf width or plant height) (Duncan’ s multiple range test *p* < 0.05).

**Supplementary Table S4 The segregation ratio of kanamycin-resistant (Kan^R^) to kanamycin-sensitive (Kan^S^**) seedlings among T1 progeny of transgenic tobacco plants.

| Line | Kan^R^ / Kan^S^ | Segregation ratio (χ^2^_0.05_ test) |
| --- | --- | --- |
| OE 2 | 78 / 27 | 2.9:1^N.S^. |
| OE 7 | 108 / 22 | 3.6:1 ^N.S^. |
| OE 9 | 88 / 27 | 3.3:1 ^N.S^. |
| *Promoter_KdN41_::GUS* 10 | 89 / 31 | 2.9:1 ^N.S^. |
| *Promoter_KdN41_::GUS* 21  *Promoter_KdN41_::GUS* 25 | 107 / 33  74 / 24 | 3.2:1 ^N.S^.  3.1:1 ^N.S^. |
| PC 1 | 84 / 25 | 3.4:1 ^N.S^. |
| PC 6 | 97 / 35 | 2.8:1 ^N.S^. |
| PC 20 | 90 / 29 | 3.1:1 ^N.S^. |
| NC 4 | 86 / 25 | 3.4:1 ^N.S^. |
| NC 8 | 93 / 35 | 2.7:1 ^N.S^. |
| NC 16 | 103 / 30 | 3.4:1 ^N.S^. |

N.S. (No significant difference) indicates no significant difference between the transgene segregation ratio and the expected 3:1 Mendelian segregation for a single insertion site (*P* > 0.05). OE: *35S::N41*, over-expression, PC: *35S::GUS*, positive control, NC: *35S::None*, negative control.

**Supplementary Table S5** Summary of stress treatments in transgenic and WT tobacco plants.

| Stress | Purpose | Transgenic plant (number) used | Control plant (number) used | Growth stage |  | Environmental condition | Treatment |
| --- | --- | --- | --- | --- | --- | --- | --- |
| Drought | Function identification | OE (9) | WT (9)  NC (9) | 3 month-old T1 generation plant |  | 22–25 °C, 16/8 h light (250 μmol m^-2^ s^-1^), 50–70% relative humidity. | Cultured in substrate (peat : perlite = 3:1, v/v).  **Light drought stress**: During week 3 after soil drying, 8-10% (8.34% in average) soil water content.  **Medium drought stress**: During week 4 after soil drying, 3-5% (3.42% in average) soil water content.  **Severe drought stress**: During week 5 after soil drying, below 1% (0.21% in average) soil water content. |
|  | GUS staining | *Promoter_KdN41_::GUS* (9) | WT (9)  PC (9) | T1 seedling |  | 22–25 °C, 16/8 h light (250 μmol m^-2^ s^-1^), 50–70% relative humidity. | Grown in 20% PEG6000 (w/v) of MS liquid medium for 10 h |
|  | Function identification | OE (9) | WT (9)  NC (9) |  |  |  |  |
| Salt | GUS staining | *Promoter_KdN41_::GUS* (45) | WT (45)  PC (45) | T1 seedling |  | 22–25 °C, 16/8 h light (250 μmol m^-2^ s^-1^), 50–70% relative humidity. | Grown on MS medium containing 400 mM NaCl for 7 days. |
|  | Function identification | OE (45) | WT (45)  NC (45) |  |  |  |  |
| Heat | GUS staining | *Promoter_KdN41_::GUS* (45) | WT (45)  PC (45) | T1 seedling |  | 16/8 h light (250 μmol m^-2^ s^-1^) | Grown on MS medium at 50 °C for 7 h in a growth chamber. |
|  | Function identification | OE (45) | WT (45)  NC (45) |  |  |  |  |

OE: *KdN41* overexpression plants; WT: wild type plants; NC: negative control plants (transformed with empty vector); PC: positive control (*35S::GUS*)

**Supplementary Table S6** Primers used in this work.

| **Primer used in RACE assay** |  |
| --- | --- |
| 3’ RACE | GSP1 5’ TGAAGGCTGGGAGACCATAGA 3’ |
|  | GSP2 5’ GCGAAGTGCGACAACAAGG 3’ |
| 5’ RACE | GSP1 5’ TCTATGGTCTCCCAGCCTTCA 3’ |
|  | GSP2 5’ GCTCCCGTCAGGCTTGAATGT 3’ |
|  |  |
| **Primer used in Genome walking assay** |  |
| SP1 5’ CTTCTATGGTCTCCCAGCCTTCA 3’ |  |
| SP2 5’ ACCTTGTTGTCGCACTTCGCTGGC 3’ |  |
| SP3 5’ TTCACCCCTGGACTCTCTATCACG 3’ |  |
|  |  |
| **Primer used in hormone treatment assay** |  |
| *KdN41* | F: 5’ ACAACAAGGTCCATCTGGAAGA 3’ |
|  | R: 5’ TGAGATTAGTCATCAGAA 3’ |
| *KdActin* | F: 5’ GACTATGAGGCTGAGTTGGAGAC 3’ |
|  | R: 5’ TCAATGAAGGCTGGAAAAGG 3’ |
|  |  |
| **Primer ued in plasmid construction** |  |
| *KdN41* gene OE expression plasmid (35S::N41) | F (BamH1): 5’ CGGGATCCATGAGGCTACATCAGTCTATAAAAG 3’ |
|  | R (Sal1): 5’ GCGTCGACGTCATCAGAAGTGGCTGCAGCCATC 3’ |
| Tissue expression plasmid (*Promoter_KdN41_::GUS* ) | F (HindIII): 5’ CCAAGCTTAGGAGTAGCATAGGACGACCACTTC 3’ |
|  | R (BamH1): 5’ CGGGATCCTTTTACTGTCCTGTAGTGTGTAAGA 3’ |
| Subcellular localization plasmid (35S::N41::YFP) | F (XbaI): 5’ GCTCTAGAATGAGGCTACATCAGTCTATAAAAG 3’ |
|  | R (BamH1): 5’ CGGGATCCGTCATCAGAAGTGGCTGCAGCCATC 3’ |
| **Primer used in positive line detection** |  |
| *Acs* (agrocinopine synthase) gene | F: 5’ AGTCAGCGATTGCAGCGGTA 3’ |
|  | R: 5’ AGTCAGCGATTGCAGCGGTA 3’ |
| *NPTII* | F: 5’ CGCTTGGGTGGAGAGGCTATTC 3’ |
|  | R: 5’ GTCCCTTCCCGCTTCAGTGACA 3’ |
| **Primer used in drought stress assay** |  |
| *NtTIR1* | F: 5’ GAAGTGGAGGACCTTAGTGGA 3’ |
|  | R: 5’ GACATCAGACCGTACATCAGC 3’ |
| *NtABP1* | F: 5’ AACGGATTACCACTTGTGAGG 3’ |
|  | R: 5’ TATGGAAGGTGCTATTAGGGA3’ |
| *NtPIN1* | F: 5’ AAAGTTGAAGGGCAAAGAAAC 3’ |
|  | R: 5’ GTCATTACACTTGTTGGAGGC 3’ |
| *NtDREB-like* | F: 5’ TTCGGACCCACTTGCTGATT 3’ |
|  | R: 5’ AAGGGAAAGTGCCAAGCCAT 3’ |
| *NtSOD* | F: 5’ GACGGACCTTAGCAACAGG 3’ |
|  | R: 5’ CTGTAAGTAGTATGCATGTTC 3’ |
| *NtCAT1* | F: 5’ TGGATCTCATACTGGTCTCA 3’ |
|  | R: 5’ TTCCATTGTTTCAGTCATTCA 3’ |
| *NtABF* | F: 5’ GAACGCGGACTTCCTGTGAC 3’ |
|  | R: 5’ TTATGCCATCAGCACCAACC 3’ |
| *NtRbohD* | F: 5’ ACCAGCACTGACCAAAGAA 3’ |
|  | R: 5’ TAGCATCACAACCACAACTA 3’ |
| *NtPOD1* | F: 5’ GTTTCTGAATACAGTAACAGCCCTCG 3’ |
|  | R: 5’ GACACTGCCACAGACTTTTCTTATGA 3’ |
| *NtEF1α* | F: 5’ CCTCTTGACCCGCAGTTACAT 3’ |
|  | R: 5’ TGATTGGTGCAGATCCCTCTA 3’ |
